# Supplementary material for: A new species of Brachycephalus (Anura: Brachycephalidae) from Serra do Quiriri, northeastern Santa Catarina state, southern Brazil, with a review of the diagnosis among species of the B. pernix group and proposed conservation measures
Source: PLoS One. 2025 Dec 10;20(12):e0334746. doi: 10.1371/journal.pone.0334746 (PMC12694819; doi:10.1371/journal.pone.0334746)
Supplement: S1 Table — (DOCX) [file pone.0334746.s001.docx]

**S1 Table. Primers used to amplify the loci used in the present study and their corresponding annealing temperatures and durations.**

| Locus | Primers | Sequence | Reference^1^ | Annealing temperature and duration |
| --- | --- | --- | --- | --- |
| 16S | 16SA–L | CGCCTGTTTATCAAAAACAT | Vences *et al.* (2000) | 56 °C, 50 s |
|  | 16SB–H | CCCGTCTGAACTCAGATCACGT |  |  |
| β-fibrinogen (β-fib) | tmFibF1 | CCAGTAGTATCTGCCATTAGGGTTA | Fitzpatrick *et al.* (2009) | 56 °C, 35 s |
|  | tmFibR1 | TTCACAATGGCATGTTCTTCA |  |  |
| L3 ribosomal protein (L3) | RPL35F | AAGAAGTCYCACCTCATGGAGAT | Pinho *et al.* (2010) | 48 °C, 40 s |
|  | RPL3RA | AGTTTCTTTGTGTGCCAACGGCTAG |  |  |
| tyrosinase exon I (Tyr) | Tyr1C | GGCAGAGGAWCRTGCCAAGATGT | Boussuyt & Milinkovitch (2000) | 62 °C, 35 s |
|  | Tyr1G | TGCTGGGCRTCTCTCCARTCCCA |  |  |

^1^References:

Boussuyt F, Milinkovitch MC. Convergent adaptive radiations in Madagascan and Asian ranid frogs reveal covariation between larval and adult traits. Proceedings of the National Academy of Sciences. 2000; 97(12):6585–90. doi: 10.1073/pnas.97.12.6585

Fitzpatrick SW, Brasileiro CA, Haddad CFB, Zamudio KR. Geographical variation in genetic structure of an Atlantic Coastal Forest frog reveals regional differences in habitat stability. Molecular Ecology. 2009;18(13):2877–96. doi: 10.1111/j.1365-294X.2009.04245.x

Pinho C, Rocha S, Carvalho BM, Lopes S, Mourão S, Vallinoto M. *et al.* New primers for the amplification and sequencing of nuclear loci in a taxonomically wide set of reptiles and amphibians. Conservation Genetics Resources. 2010;2:181–5. doi: 10.1007/s12686-009-9126-4

Vences MKJ, Lötters S, Widmer A, Jungfer K, Köhler J, Veith M. Phylogeny and classification of poison frogs (Amphibia: Dendrobatidae), based on mitochondrial 16S and 12S ribosomal RNA gene sequences. Molecular Phylogenetics and Evolution. 2000;15:34–40. doi: 10.1006/mpev.1999.0738
