# Supplementary material for: A new species of Brachycephalus (Anura: Brachycephalidae) from Serra do Quiriri, northeastern Santa Catarina state, southern Brazil, with a review of the diagnosis among species of the B. pernix group and proposed conservation measures
Source: PLoS One. 2025 Dec 10;20(12):e0334746. doi: 10.1371/journal.pone.0334746 (PMC12694819; doi:10.1371/journal.pone.0334746)
Supplement: S2 Table — (DOCX) [file pone.0334746.s002.docx]

**S2 Table. GenBank accession numbers of all sequences used in our analyses, as well as the new sequences generated in the present study.**

| Species | 16S | β-fib | L3 | Tyr |
| --- | --- | --- | --- | --- |
| *Brachycephalus quiririensis* 1 | KX025317; https://www.ncbi.nlm.nih.gov/nuccore/KX025317 | KX025858; https://www.ncbi.nlm.nih.gov/nuccore/KX025858 | KX026461; https://www.ncbi.nlm.nih.gov/nuccore/KX026461 | KX026163; https://www.ncbi.nlm.nih.gov/nuccore/KX026163 |
| *Brachycephalus quiririensis* 2 | KX025318; https://www.ncbi.nlm.nih.gov/nuccore/KX025318 | KX025859; https://www.ncbi.nlm.nih.gov/nuccore/KX025859 | KX026462; https://www.ncbi.nlm.nih.gov/nuccore/KX026462 | KX026164; https://www.ncbi.nlm.nih.gov/nuccore/KX026164 |
| *Brachycephalus quiririensis* 3 | KX025319; https://www.ncbi.nlm.nih.gov/nuccore/KX025319 | KX025860; https://www.ncbi.nlm.nih.gov/nuccore/KX025860 | KX026463; https://www.ncbi.nlm.nih.gov/nuccore/KX026463 | KX026165; https://www.ncbi.nlm.nih.gov/nuccore/KX026165 |
| *Brachycephalus quiririensis* 4 | KX025320; https://www.ncbi.nlm.nih.gov/nuccore/KX025320 | KX025861; https://www.ncbi.nlm.nih.gov/nuccore/KX025861 | KX026464; https://www.ncbi.nlm.nih.gov/nuccore/KX026464 | KX026166; https://www.ncbi.nlm.nih.gov/nuccore/KX026166 |
| *Brachycephalus quiririensis* 5 | KX025321; https://www.ncbi.nlm.nih.gov/nuccore/KX025321 | KX025862; https://www.ncbi.nlm.nih.gov/nuccore/KX025862 | KX026465; https://www.ncbi.nlm.nih.gov/nuccore/KX026465 | KX026167; https://www.ncbi.nlm.nih.gov/nuccore/KX026167 |
| *Brachycephalus quiririensis* 6 | KX025322; https://www.ncbi.nlm.nih.gov/nuccore/KX025322 | KX025863; https://www.ncbi.nlm.nih.gov/nuccore/KX025863 | KX026466; https://www.ncbi.nlm.nih.gov/nuccore/KX026466 | KX026168; https://www.ncbi.nlm.nih.gov/nuccore/KX026168 |
| *Brachycephalus quiririensis* 7 | ^TAAGAGGTCTAGCCTGCCCAGTGACTAAAATTCAACGGCCGCGGTATCCTGACCGCGCGAAGGTAGCGTAATCACTTGTTTTTTAAATAAAGACTAGTATGAATGGCACCACGAGGATCAAACTGTCTCCTTTACATAATCAGTGAAACTAATCCCCCCGTGAAAAAGCAGGGATTAAACTACAAGACGAAAAGACCCTATGGAGCTTTAAATATGTAACTAGCACTAGTTATAAGTTTTAGGTTGGGGTGACCGCGGAGAAAAACATATCCTCCATAACGAATAAGTTTACTTTAAACAAAAAGTCACAACTTCACGTATCAACAAATTGACGTACTGATCCAATAATCCGATCAACGAACCAAGTTACCCTAGGGATAACAGCGCAATCCACTTTAAGAGTTCATATCGACAAGTGGGCTTACGACCTCGATGTTGGATCAGGGTATCCCAGTGGTGCAGCCGCTACTAAAG^ | ^ATGTTCTTCAGTACTTATGACAGAGACAATGATGGCTGGTATGTATGGAACTCGCCCTTCATTTCTTATGCTGCATGTCCATTGGTGATTTAGAGCCTCACGCTGACTCANNNAACATTTCCGAGACAGTTAATAACTATTAAATTGTTGCCTTGCCTAAAGTCACATTCATCCTTAGATAGTGGTTTAGATTGTACCTTATGAATCTAACCTTTACTGAAGTTTCCTACCCCAGTAAGTCAAAGGGAAACAGACCAGACCCATATGACAGTGTCCCCTCTTTGCGAGTCTTGATAGTCTTATAATCGATAATGGTCATGTTTTCAGACTCATTAGAAGTAATGTTTTATTTTCTAGGTCACATGC^ | – | – |
| *Brachycephalus pombali* | KX025310; https://www.ncbi.nlm.nih.gov/nuccore/KX025310 | KX025851; https://www.ncbi.nlm.nih.gov/nuccore/KX025851 | KX026454; https://www.ncbi.nlm.nih.gov/nuccore/KX026454 | KX026156; https://www.ncbi.nlm.nih.gov/nuccore/KX026156 |
| *Brachycephalus ferruginus* | KX025236; https://www.ncbi.nlm.nih.gov/nuccore/KX025236 | KX025777; https://www.ncbi.nlm.nih.gov/nuccore/KX025777 | – | KX026082; https://www.ncbi.nlm.nih.gov/nuccore/KX026082 |
| *Brachycephalus auroguttatus* | KX025354; https://www.ncbi.nlm.nih.gov/nuccore/KX025354 | KX025895; https://www.ncbi.nlm.nih.gov/nuccore/KX025895 | KX026492; https://www.ncbi.nlm.nih.gov/nuccore/KX026492 | KX026200; https://www.ncbi.nlm.nih.gov/nuccore/KX026200 |
| *Brachycephalus pernix* | KX025218; https://www.ncbi.nlm.nih.gov/nuccore/KX025218 | KX025759; https://www.ncbi.nlm.nih.gov/nuccore/KX025759 | KX026365; https://www.ncbi.nlm.nih.gov/nuccore/KX026365 | KX026064; https://www.ncbi.nlm.nih.gov/nuccore/KX026064 |
| *Brachycephalus lulai* 1 | ^TAAGAGGTCTAGCCTGCCCAGTGACTAAAATTCAACGGCCGCGGTATCCTGACCGCGCGAAGGTAGCGTAATCACTTGTTTTTTAAATAAAGACTAGTATGAATGGCACCACGAGGATCAAACTGTCTCCTTTACATAATCAGTGAAACTAATCTCCCCGTGAAAAAGCAGGGATTAAACTACAAGACGAAAAGACCCTATGGAGCTTTAAATATGTAACTAGCACTAGTTATAAGTTTTAGGTTGGGGTGACCGCGGAGAAAAACATATCCTCCATGACGAATAAGTTTACTTTAAACAAAAAGTCACAACTTCATGTATCAACAAATTGACATACTGATCCAATAATCTGATCAACGAACCAAGTTACCCTAGGGATAACAGCGCAATCCACTTTAAGAGTTCATATCGACAAGTGGGCTTACGACCTCGATGTTGGATCAGGGTATCCCAGTGGTGC^ | – | – | ^GCCAAGATGTTGTCGTATCTACCTCGCCCTTGGGTCCTCAATTTCCCTTTAGTGGGATTGACGATAGGGAAAACTGGCCTATTGTCTTCTACAACAGGACCTGTCAATGCGGGGGTAACTTCATGGGCTACAATTGCGGCGACTGCAAGTTTGGTTTCATTGGCCCCAACTGTACGGTGAGAAGAACGATGATCAGGAAAGAGATCTTCAAGATGACTTCGGCGGAGAAGGATAAGTTAATTGCCTACCTCAATCTGGCAAAACGCACCATCAGCCTGGACTACGTCATCGCCACCGGGACTTACGAGCAAATGAACAATGGCTCCAATCCACTTTTTGCGGACATTAGCGTGTACGACCTGTTTGTGTGGCTGCACTATTACTCCTCCCGGGACGCTTTCCTAGAAGGTGACCTTGTGTGGAGAGATATTGACTTTGCCCACGAAGCACCAGCTTTTTTGCCCTGGCATAGGTTTTTCTTGCTTCACTGGGAACATGAAATTCAGAAGCTTACCGGTGATGAGAACTTCACCATTCCTTTCTGGGACTGGAGAGACGCCCAGCAA^ |
| *Brachycephalus lulai* 2 | ^TAAGAGGTCTAGCCTGCCCAGTGACTAAAATTCAACGGCCGCGGTATCCTGACCGCGCGAAGGTAGCGTAATCACTTGTTTTTTAAATAAAGACTAGTATGAATGGCACCACGAGGATCAAACTGTCTCCTTTACATAATCAGTGAAACTAATCTCCCCGTGAAAAAGCAGGGATTAAACTACAAGACGAAAAGACCCTATGGAGCTTTAAATATGTAACTAGCACTAGTTATAAGTTTTAGGTTGGGGTGACCGCGGAGAAAAACATATCCTCCATGACGAATAAGTTTACTTTAAACAAAAAGTCACAACTTCATGTATCAACAAATTGACATACTGATCCAATAATCTGATCAACGAACCAAGTTACCCTAGGGATAACAGCGCAATCCACTTTAAGAGTTCATATCGACAAGTGGGCTTACGACCTCGATGTTGGATCAGGGTATCCCAGTGGTGCAGCCGCTACTAAAG^ | ^AGCCGCTACTAAAGATGTTCTTCAGTACTTATGACAGAGACAATGATGGCTGGTATGTATGGAACTCGCCCTTCATTTCTTATGCTGCATGTCCATTGGTGATTTAGAGCCTCACGCTGACTCANNNAACATTTCCGAGACAGTTAATAACTATTAAATTGTCGCCTTGCCTAAWGTCACATTCATCCTTAGATAGTGGTTTAGATTGTACCTTATGAATCTAACCTTTACTGAAGTTTCCTACCCCAGTAAGTCAAAGGGAAACAGACAAGACCCATATGACAGTGTCCCCTCTTTGCGAGTCTTGATAGTCTTATAATCGATAATGGTCATGTTTTCAGACTCATTAGAAGTAATGTTTTATTTTCTAGGTCACATGCAGATCCAAACAAACAGTGC^ | ^GCCAAAGAAGACGGNNNGAAGTCTCACCTCATGGAGATCCAAGTGAACGGAGGGACAATTGCCGAGAAGTTGGACTGGGCCCGTGAGAAGCTGGAGCAGCAGGTGGCAGTGTCTGGAGTCTTTGGTCAAGATGAAATGATAGATGTCATTGGAGTCACAAAGGGAAAAGGCTACAAAGGTGAGACTGATGGGCTTTTTCTGTAACAAAGTTCCCTTACGTTAACAGCCCATGAGTAGTGTGTAGAGACGTTAGGACTGTTTAGTCTGTTCTGTGATGAGGTCATGGAATAAGCACTGGGCACAGTGCCTGAATAATACTGAAGATTAATTCCATGCTGCCTTCCTTCTGAGAACAAGTTTGTAGTAAGGTCACTAAATGGAGCTGTGTTG^ | ^GTTGTTTCTTAATGCCAAGATGTTGTCGTATCTACCTCGCCCTTGGGTCCTCAATTTCCCTTTAGTGGGATTGACGATAGGGAAAACTGGCCTATTGTCTTCTACAACAGGACCTGTCAATGCCGGGGTAACTTCATGGGCTACAATTGCGGCGACTGCAAGTTTGGTTTCATTGGCCCCAACTGTACGGTGAGAAGAACGATGATCAGGAAAGAGATATTCAAGATGACTTCGGCGGAGAAGGATAAGTTAATTGCCTACCTCAATCTGGCAAAACGCACCATCAGCCTGGACTACGTCATCGCCACCGGGACTTACGAGCAAATGAACAATGGCTCCAATCCACTTTTTGCGGACATTAGCGTGTACGACCTGTTTGTGTGGCTGCACTATTACTCCTCCCGGGACGCTTTCCTAGAAGGTGACCTCGTGTGGAGAGATATTGACTTTGCCCACGAAGCACCAGCTTTTTTGCCCTGGCATAGGTTTTTCTTGCTTCACTGGGAACATGAAATTCAGAAGCTTACCGGTGATGAGAACTTCACCATTCCTTTCTGGGACTGGAGAGACGCCCAGCAA^ |
| *Brachycephalus lulai* 3 | ^TAAGAGGTCTAGCCTGCCCAGTGACTAAAATTCAACGGCCGCGGTATCCTGACCGCGCGAAGGTAGCGTAATCACTTGTTTTTTAAATAAAGACTAGTATGAATGGCACCACGAGGATCAAACTGTCTCCTTTACATAATCAGTGAAACTAATCTCCCCGTGAAAAAGCAGGGATTAAACTACAAGACGAAAAGACCCTATGGAGCTTTAAATATGTAACTAGCACTAGTTATAAGTTTTAGGTTGGGGTGACCGCGGAGAAAAACATATCCTCCATGACGAATAAGTTTACTTTAAACAAAAAGTCACAACTTCATGTATCAACAAATTGACATACTGATCCAATAATCTGATCAACGAACCAAGTTACCCTAGGGATAACAGCGCAATCCACTTTAAGAGTTCATATCGACAAGTGGGCTTACGACCTCGATGTTGGATCAGGGTATCCCAGTGGTGCAGCCGCTACTAAAG^ | – | – | ^CCAAGATGTTGTCGTATCTACCTCGCCCTTGGGTCCTCAATTTCCCTTTAGTGGGATTGACGATAGGGAAAACTGGCCTATTGTCTTCTACAACAGGACCTGTCAATGCCGGGGTAACTTCATGGGCTACAATTGCGGCGACTGCAAGTTTGGTTTCATTGGCCCCAACTGTACGGTGAGAAGAACGATGATCAGGAAAGAGATCTTCAAGATGACTTCGGCGGAGAAGGATAAGTTAATTGCCTACCTCAATCTGGCAAAACGCACCATCAGCCTGGACTACGTCATCGCCACCGGGACTTACGAGCAAATGAACAATGGCTCCAATCCACTTTTTGCGGACATTAGCGTGTACGACCTGTTTGTGTGGCTGCACTATTACTCCTCCCGGGACGCWTTCCTAGAAGGTGACCTTGTGTGGAGAGATATTGACTTTGCCCACGAAGCACCAGCTTTTTTGCCCTGGCATAGGTTTTTCTTGCTTCACTGGGAACATGAAATTCAGAAGCTTACCGGTGATGAGAACTTCACCATTCCTTTCTGGGACTGGA^ |
| *Brachycephalus lulai* 4 | ^TAAGAGGTCTAGCCTGCCCAGTGACTAAAATTCAACGGCCGCGGTATCCTGACCGCGCGAAGGTAGCGTAATCACTTGTTTTTTAAATAAAGACTAGTATGAATGGCACCACGAGGATCAAACTGTCTCCTTTACATAATCAGTGAAACTAATCTCCCCGTGAAAAAGCAGGGATTAAACTACAAGACGAAAAGACCCTATGGAGCTTTAAATATGTAACTAGCACTAGTTATAAGTTTTAGGTTGGGGTGACCGCGGAGAAAAACATATCCTCCATGACGAATAAGTTTACTTTAAACAAAAAGTCACAACTTCATGTATCAACAAATTGACATACTGATCCAATAATCTGATCAACGAACCAAGTTACCCTAGGGATAACAGCGCAATCCACTTTAAGAGTTCATATCGACAAGTGGGCTTACGACCTCGATGTTGGATCAGGGTATCCCAGTGGTGCAGCCGCTACTAAAG^ | ^CAGCCGCTACTAAAGATGTTCTTCAGTACTTATGACAGAGACAATGATGGCTGGTATGTATGGAACTCGCCCTTCATTTCTTATGCTGCATGTCCATTGGTGATTTAGAGCCTCACGCTGACTCANNNAACATTTCCGAGACAGTTAATAACTATTAAATTGTCGCCTTGCCTAAAGTCACATTCATCCTTAGATAGTGGTTTAGATTGTACCTTATGAATCTAACCTTTACTGAAGTTTCCTACCCCAGTAAGTCAAAGGGAAACAGACAAGACCCATATGACAGTGTCCCCTCTTTGCGAGTCTTGATAGTCTTATAATCGATAATGGTCATGTTTTCAGACTCATTAGAAGTAATGTTTTATTTTCTAGGTCACATGCAGATCCAAACAAACAGTGC^ | ^CCAAAGAAGACGGNNNGAAGTCTCACCTCATGGAGATCCAGGTGAACGGAGGGACAATTGCCGAGAAGTTGGACTGGGCCCGTGAGAAGCTGGAGCAGCAGGTGGCAGTGTCTGGAGTCTTTGGTCAAGATGAAATGATAGATGTCATTGGAGTCACAAAGGGAAAAGGCTACAAAGGTGAGACTGATGGGCTTTTTCTGTAACAAAGTTCCCTTACGTTAGCAGCCCATGAGTAGTGTGTAGAGACGTTGGGACTGTTTAGTCTGTTCTGTGATGAGGTCATGGAATAAGCACTGGGCACAGTGCCTGAATAATACTGAAGAATAATTCCATGCTGCCTTCCTTCTGAGAACAAGTTTGTAGTAAGGTCACTAAATGAAGCTGTGTTG^ | ^GTTGTTTCTTAATGCCAAGATGTTGTCATATCTACCTCGCCCTTGGGTCCTCAATTTCCCTTTAGTGGGATTGACGATAGGGAAAACTGGCCTATTGTCTTCTACAACAGGACCTGTCAATGCGGGGGTAACTTCATGGGCTACAATTGCGGCGACTGCAAGTTTGGTTTCATTGGCCCCAACTGTACGGTGAGAAGAACGATGATCAGGAAAGAGATCTTCAAGATGACTTCGGCGGAGAAGGATAAGTTAATTGCCTACCTCAATCTGGCAAAACGCACCATCAGCCTGGACTACGTCATCGCCACCGGGACTTACGAGCAAATGAACAATGGCTCCAACCCACTTTTTGCGGACATTAGCGTGTACGACCTGTTTGTGTGGCTGCACTATTACTCCTCCCGGGACGCTTTCCTAGAAGGTGACCTTGTGTGGAGAGATATTGACTTTGCCCACGAAGCACCAGCTTTTTTGCCCTGGCATAGGTTTTTCTTGCTTCACTGGGAACATGAAATTCAGAAGCTTACCGGTGATGAGAACTTCACCATTCCCTTCTGGGACTGGAGAGACGCCCAGCAA^ |
