# Supplementary material for: A new species of Brachycephalus (Anura: Brachycephalidae) from Serra do Quiriri, northeastern Santa Catarina state, southern Brazil, with a review of the diagnosis among species of the B. pernix group and proposed conservation measures
Source: PLoS One. 2025 Dec 10;20(12):e0334746. doi: 10.1371/journal.pone.0334746 (PMC12694819; doi:10.1371/journal.pone.0334746)
Supplement: S3 Table — (DOCX) [file pone.0334746.s003.docx]

**S3 Table. Minimum and maximum pairwise genetic distance (%) matrix for 16S sequences among specimens of *Brachycephalus* in the present study.**

| Species | *B. quiririensis* | | *B. lulai* | | *B. pombali* | | *B. pernix* | | *B. ferruginus* | |
| --- | --- | --- | --- | --- | --- | --- | --- | --- | --- | --- |
|  | Min | Max | Min | Max | Min | Max | Min | Max | Min | Max |
| *B. lulai* | 0.0065 | 0.0099 | **---** | **---** |  |  |  |  |  |  |
| *B. pombali* | 0.0064 | 0.0077 | 0.0062 | 0.0084 | **---** | **---** |  |  |  |  |
| *B. pernix* | 0.0077 | 0.0089 | 0.0075 | 0.0097 | 0.0038 | 0.0038 | **---** | **---** |  |  |
| *B. ferruginus* | 0.0074 | 0.0086 | 0.0072 | 0.0094 | 0.0035 | 0.0035 | 0.0007 | 0.0007 | **---** | **---** |
| *B. auroguttatus* | 0.0054 | 0.0094 | 0.0060 | 0.0082 | 0.0060 | 0.0060 | 0.0072 | 0.0072 | 0.0069 | 0.0069 |
