# Supplementary figures and images for: A new species of Brachycephalus (Anura: Brachycephalidae) from Serra do Quiriri, northeastern Santa Catarina state, southern Brazil, with a review of the diagnosis among species of the B. pernix group and proposed conservation measures
Source: PLoS One. 2025 Dec 10;20(12):e0334746. doi: 10.1371/journal.pone.0334746 (PMC12694819; doi:10.1371/journal.pone.0334746)

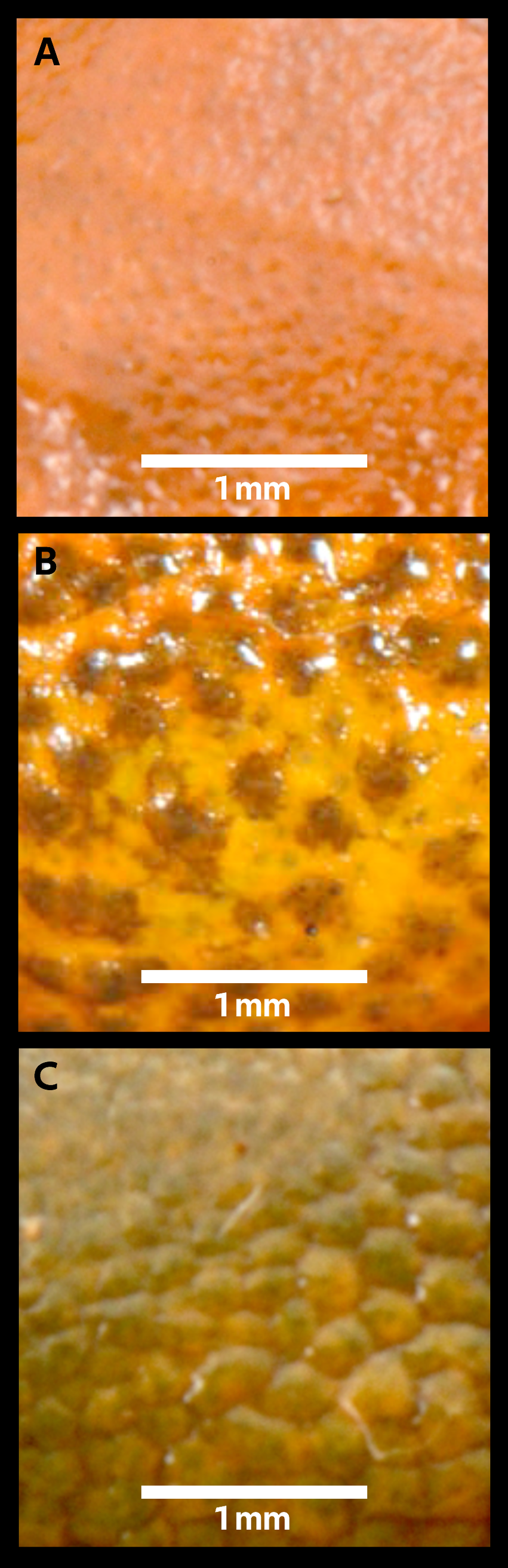

Supplement: S1 Fig — Smooth texture. B. Moderately rough texture. C. Densely rough texture. Scale bar equal 1 mm. (TIF) [file pone.0334746.s004.tif]

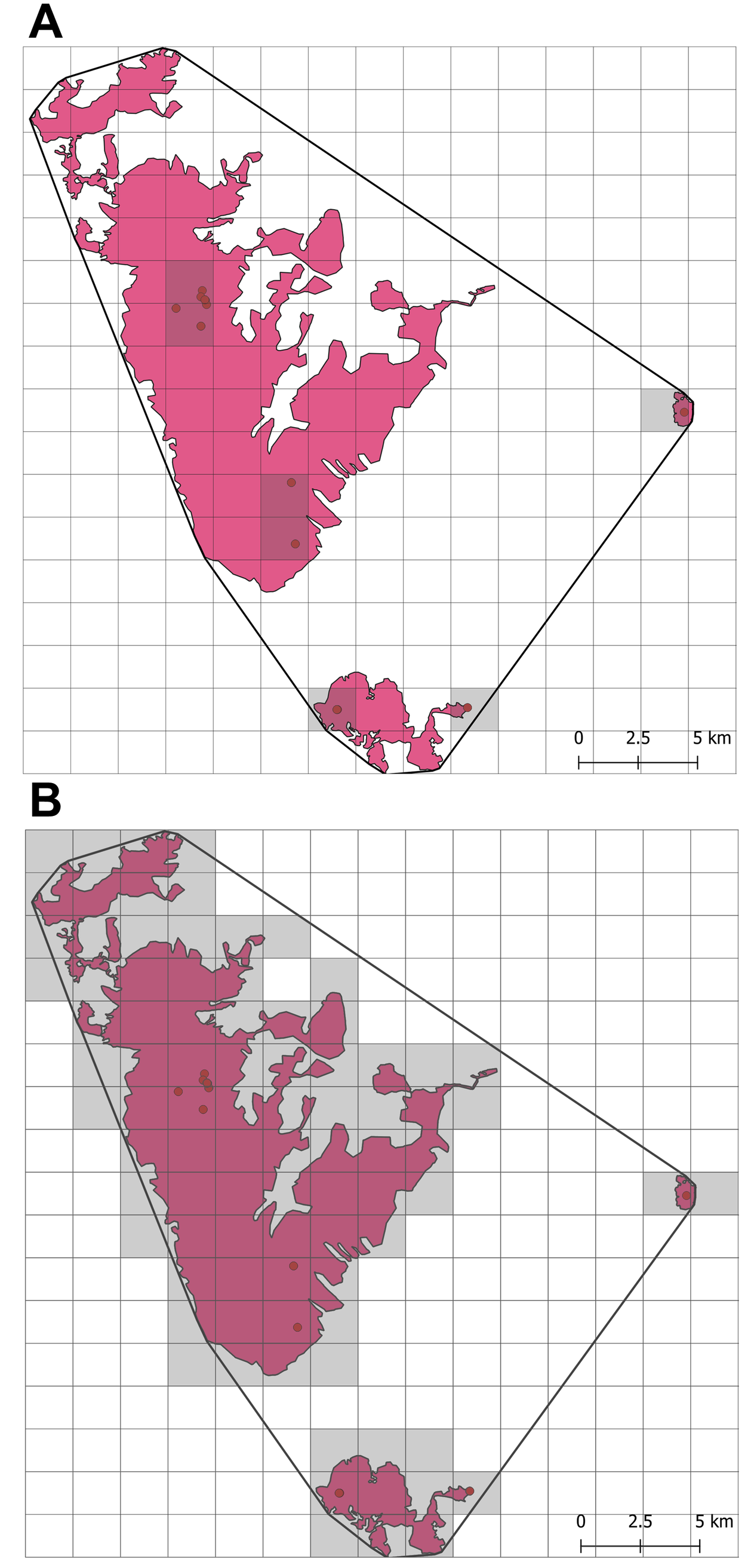

Supplement: S2 Fig — (A) Distribution considering the lower bound of area of occupancy (AOO) based on current records. (B) Distribution considering the upper bound of AOO incorporating suitable habitat. The black line represents the minimum convex polygon (MCP) of the extent of occurrence (EOO), pink polygons indicate mapped suitable habitat, red dots represent current records, and dark-shaded cells were accounted for in the estimation. All layers were created by the authors. Distribution was generated using field observations, habitat mapping, and altitude. No copyrighted or third-party material was used for the figure. (TIF) [file pone.0334746.s005.tif]

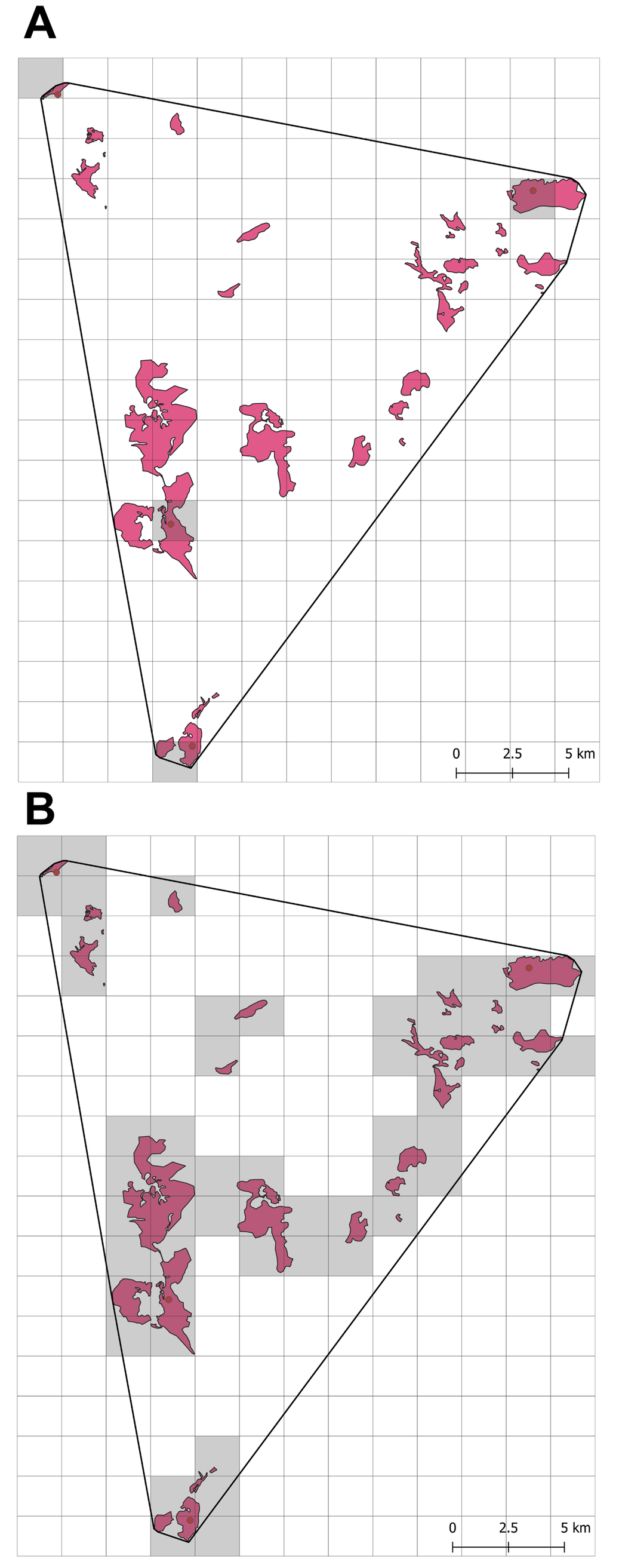

Supplement: S3 Fig — (A) Distribution considering the lower bound of area of occupancy (AOO) based on current records. (B) Distribution considering the upper bound of AOO incorporating suitable habitat. The black line represents the minimum convex polygon (MCP) of the extent of occurrence (EOO), pink polygons indicate mapped suitable habitat, red dots represent current records, and dark-shaded cells were accounted for in the estimation. All layers were created by the authors. Distribution was generated using field observations, habitat mapping, and altitude. No copyrighted or third-party material was used for the figure. (TIF) [file pone.0334746.s006.tif]

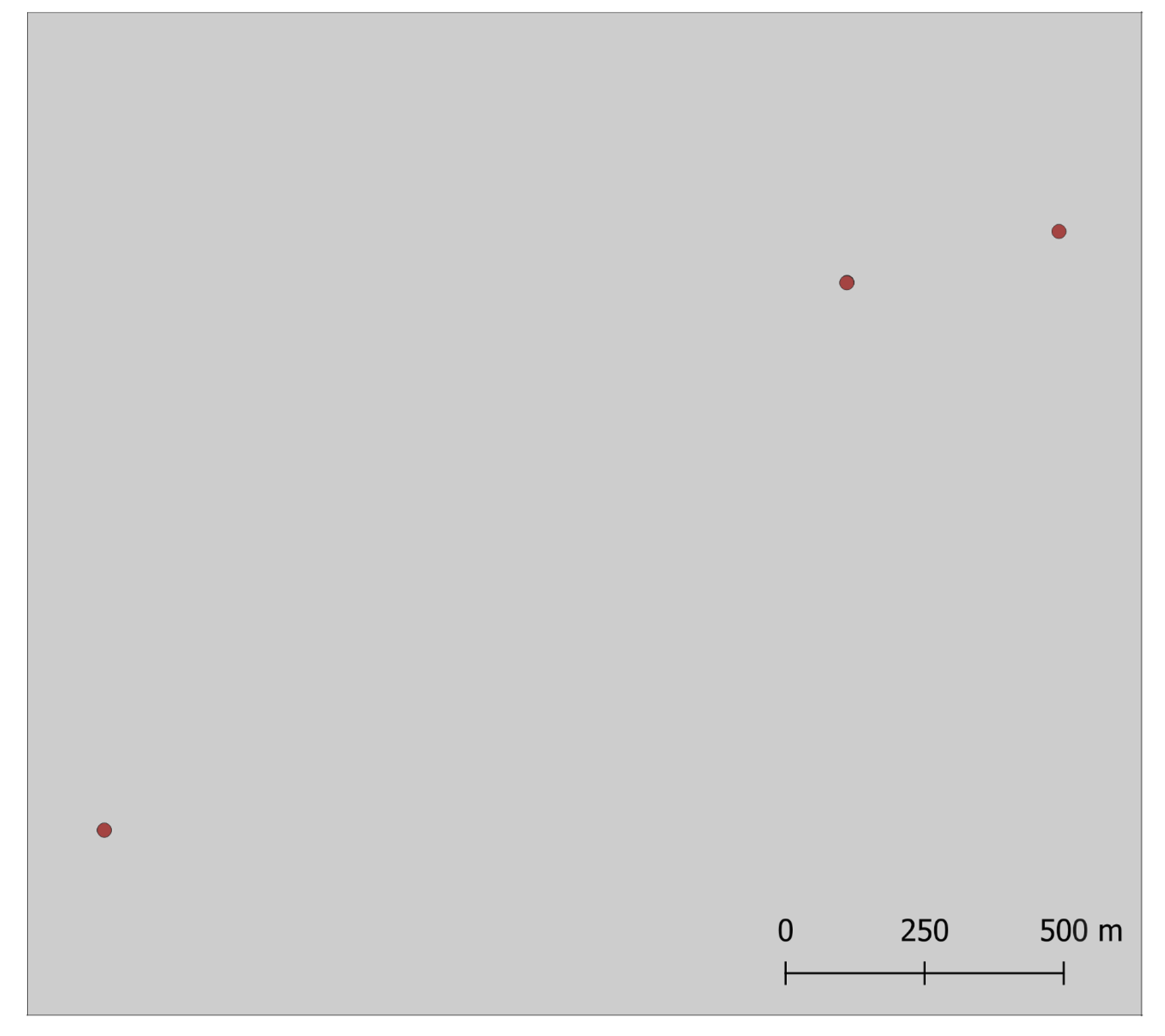

Supplement: S4 Fig — Red dots represent current records, and the dark-shaded area was accounted for in the estimation. All layers were created by the authors. Distribution was generated using field observations, habitat mapping, and altitude. No copyrighted or third-party material was used for the figure. (TIF) [file pone.0334746.s007.tif]

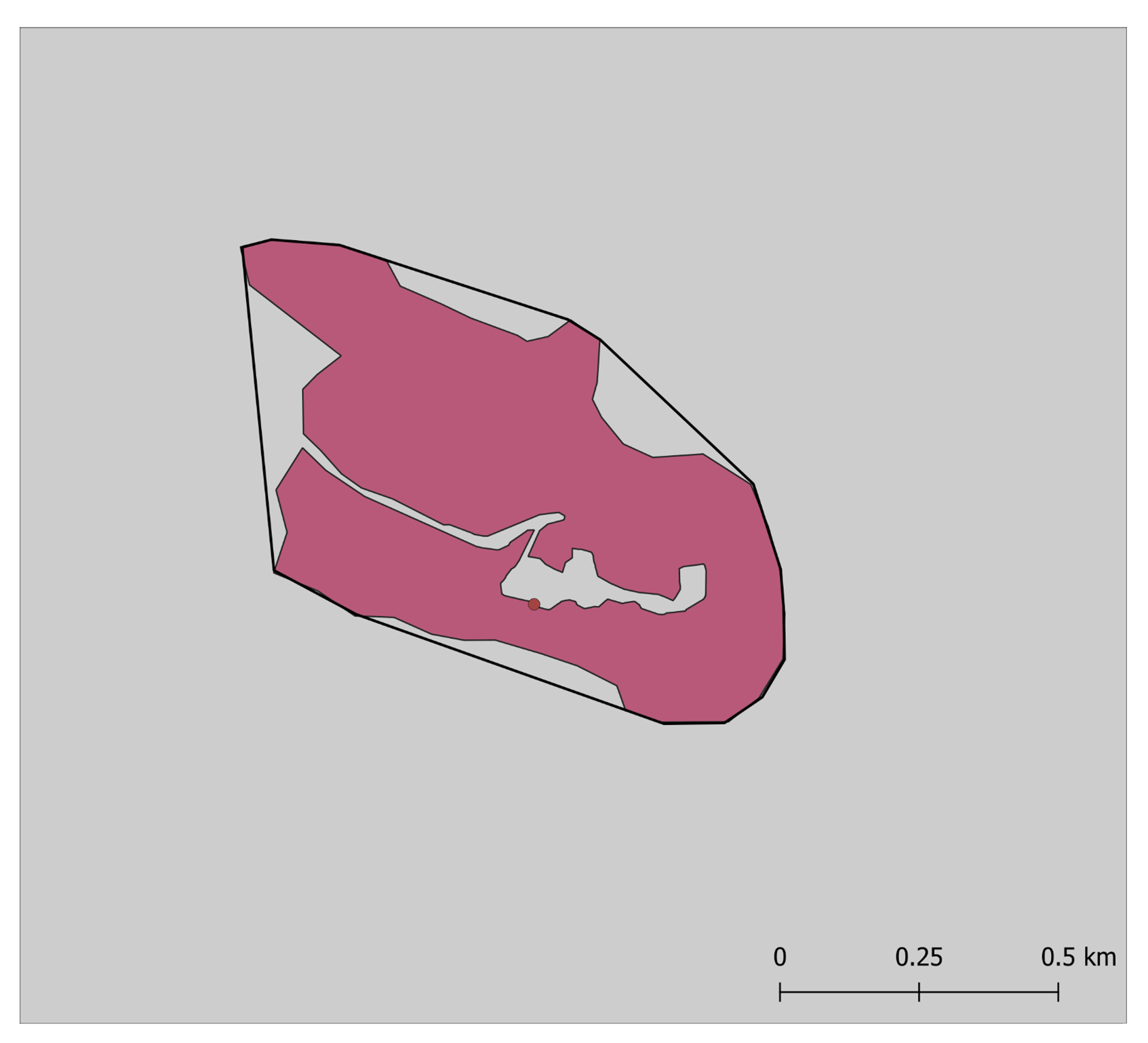

Supplement: S5 Fig — The black line represents the minimum convex polygon (MCP) of the extent of occurrence (EOO), the pink polygon indicates mapped suitable habitat, the red dot represents the current record, and the dark-shaded area was accounted for in the estimation. All layers were created by the authors. Distribution was generated using field observations, habitat mapping, and altitude. No copyrighted or third-party material was used for the figure. (TIF) [file pone.0334746.s008.tif]

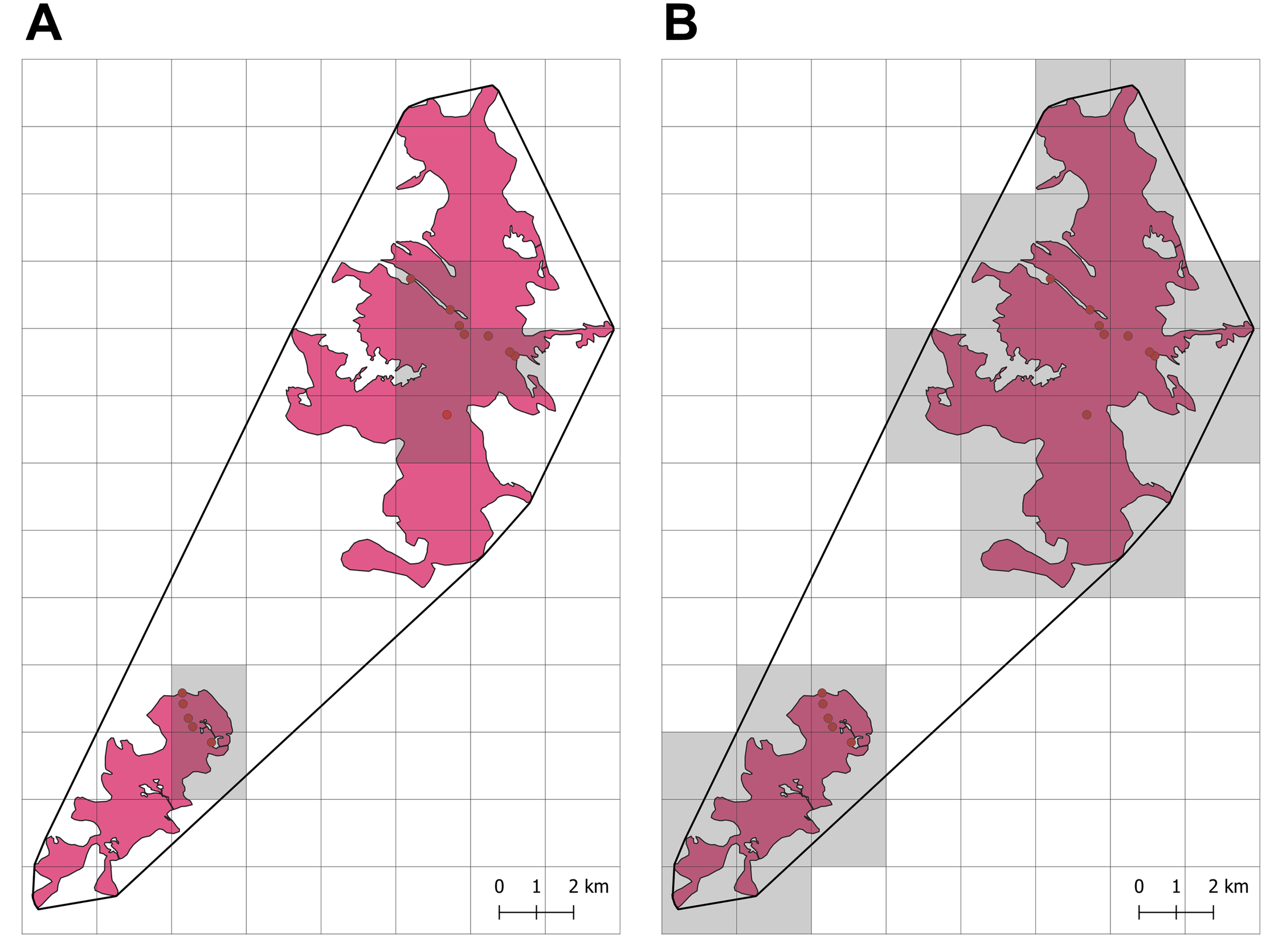

Supplement: S6 Fig — (A) Distribution considering the lower bound of area of occupancy (AOO) based on current records. (B) Distribution considering the upper bound of AOO incorporating suitable habitat. The black line represents the minimum convex polygon (MCP) of the extent of occurrence (EOO), pink polygons indicate mapped suitable habitat, red dots represent current records, and dark-shaded cells were accounted for in the estimation. All layers were created by the authors. Distribution was generated using field observations, habitat mapping, and altitude. No copyrighted or third-party material was used for the figure. (TIF) [file pone.0334746.s009.tif]

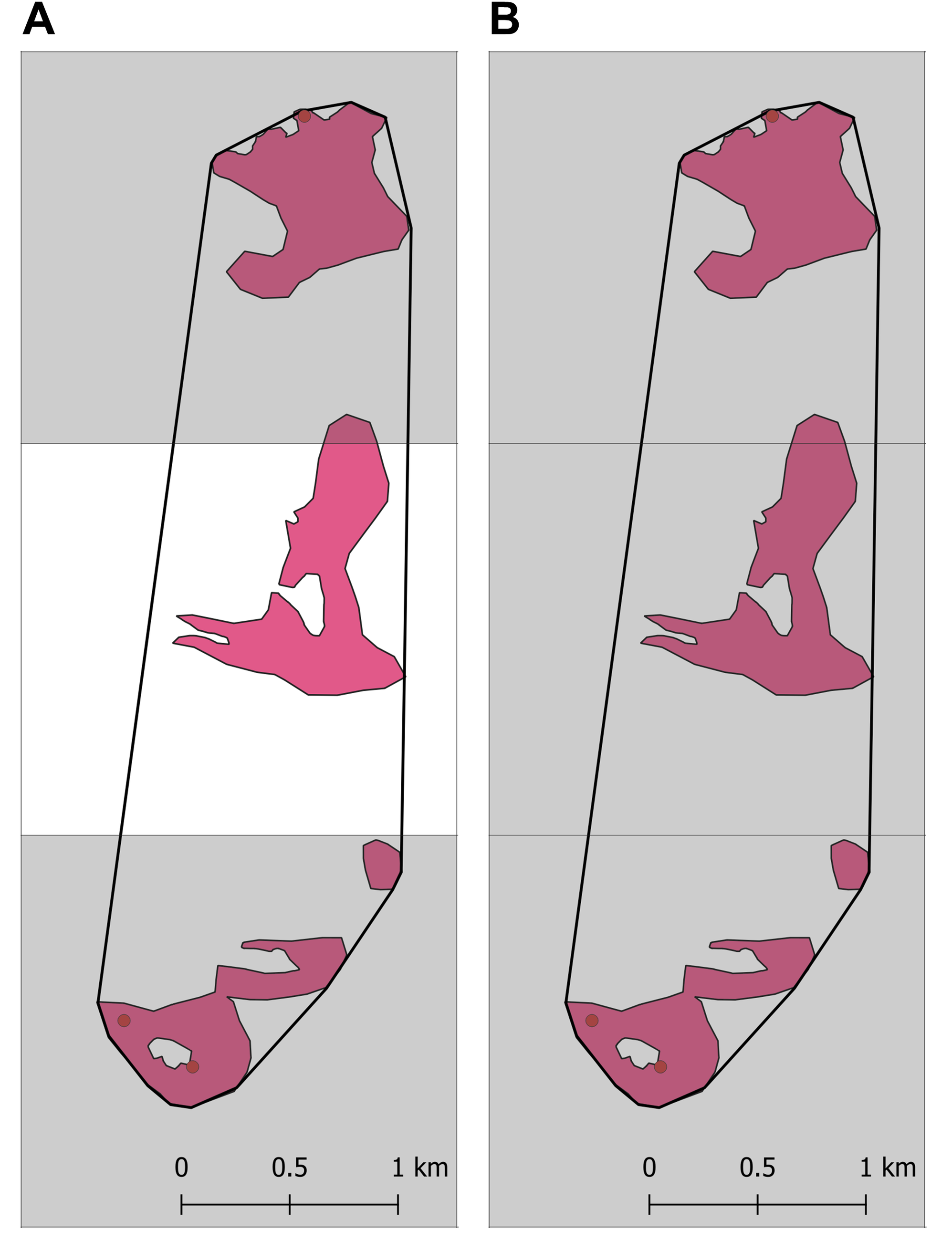

Supplement: S7 Fig — (A) Distribution considering the lower bound of area of occupancy (AOO) based on current records. (B) Distribution considering the upper bound of AOO incorporating suitable habitat. The black line represents the minimum convex polygon (MCP) of the extent of occurrence (EOO), pink polygons indicate mapped suitable habitat, red dots represent current records, and dark-shaded cells were accounted for in the estimation. All layers were created by the authors. Distribution was generated using field observations, habitat mapping, and altitude. No copyrighted or third-party material was used for the figure. (TIF) [file pone.0334746.s010.tif]

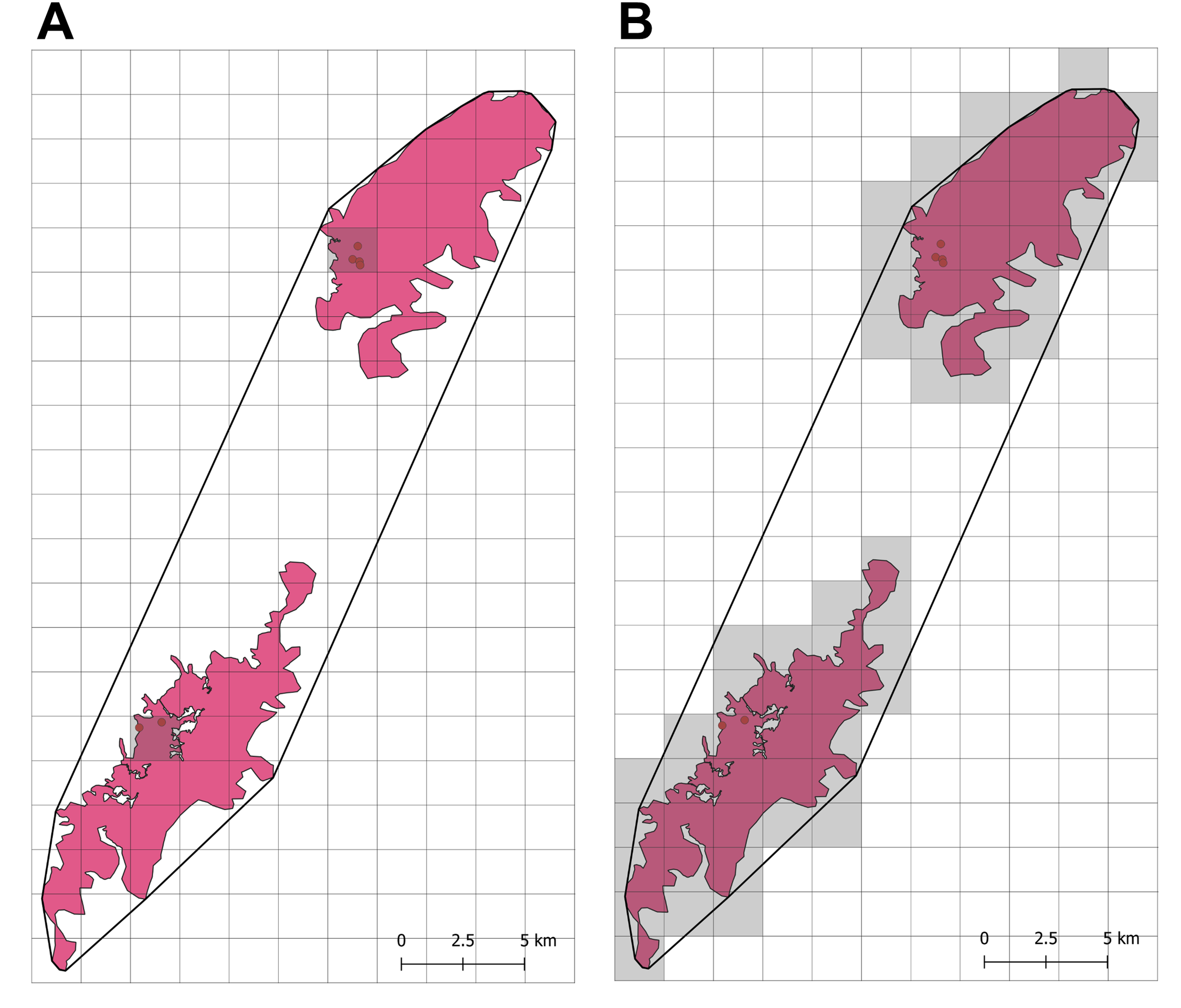

Supplement: S8 Fig — (A) Distribution considering the lower bound of area of occupancy (AOO) based on current records. (B) Distribution considering the upper bound of AOO incorporating suitable habitat. The black line represents the minimum convex polygon (MCP) of the extent of occurrence (EOO), pink polygons indicate mapped suitable habitat, red dots represent current records, and dark-shaded cells were accounted for in the estimation. All layers were created by the authors. Distribution was generated using field observations, habitat mapping, and altitude. No copyrighted or third-party material was used for the figure. (TIF) [file pone.0334746.s011.tif]

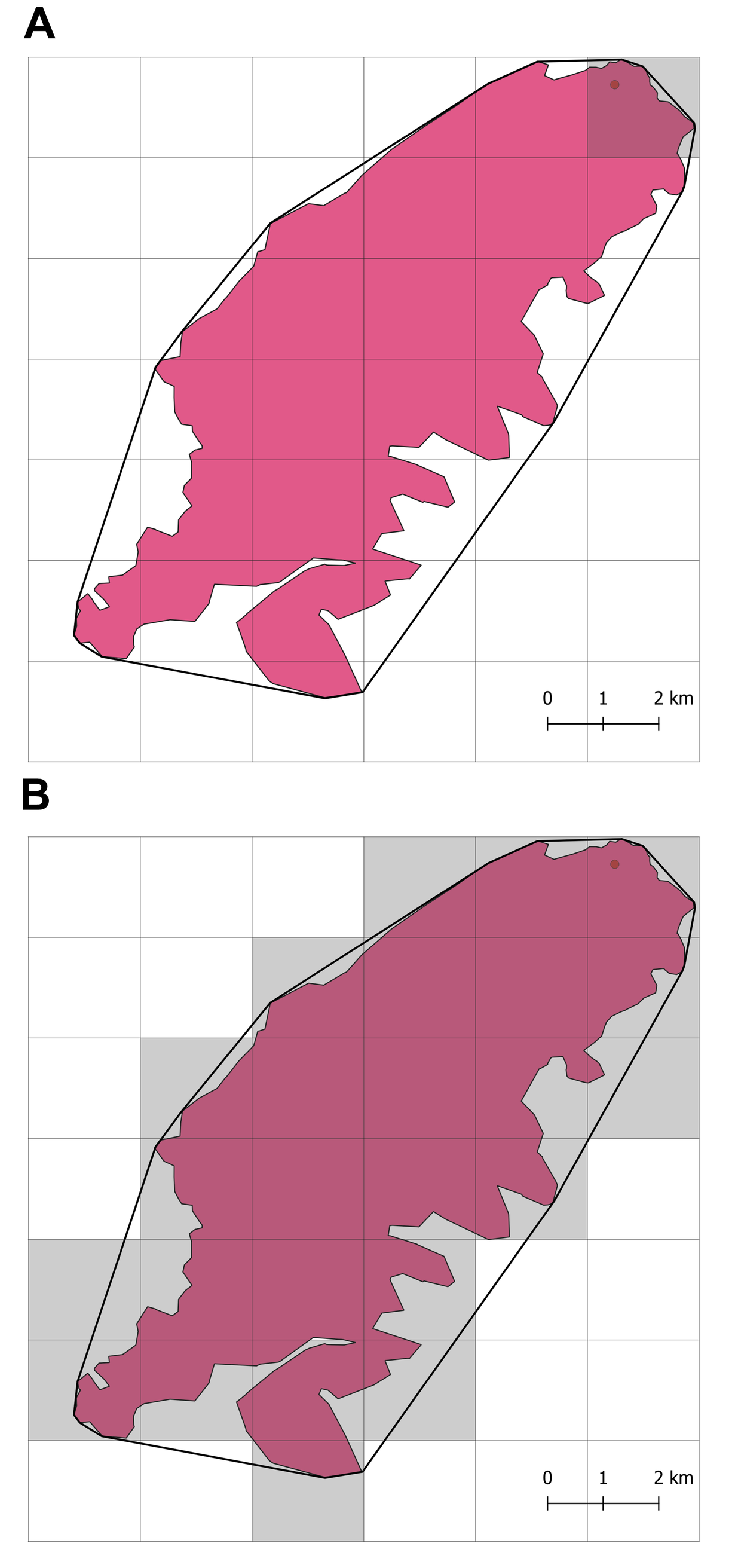

Supplement: S9 Fig — (A) Distribution considering the lower bound of area of occupancy (AOO) based on current records. (B) Distribution considering the upper bound of AOO incorporating suitable habitat. The black line represents the minimum convex polygon (MCP) of the extent of occurrence (EOO), pink polygons indicate mapped suitable habitat, the red dot represents the current record, and dark-shaded cells were accounted for in the estimation. All layers were created by the authors. Distribution was generated using field observations, habitat mapping, and altitude. No copyrighted or third-party material was used for the figure. (TIF) [file pone.0334746.s012.tif]

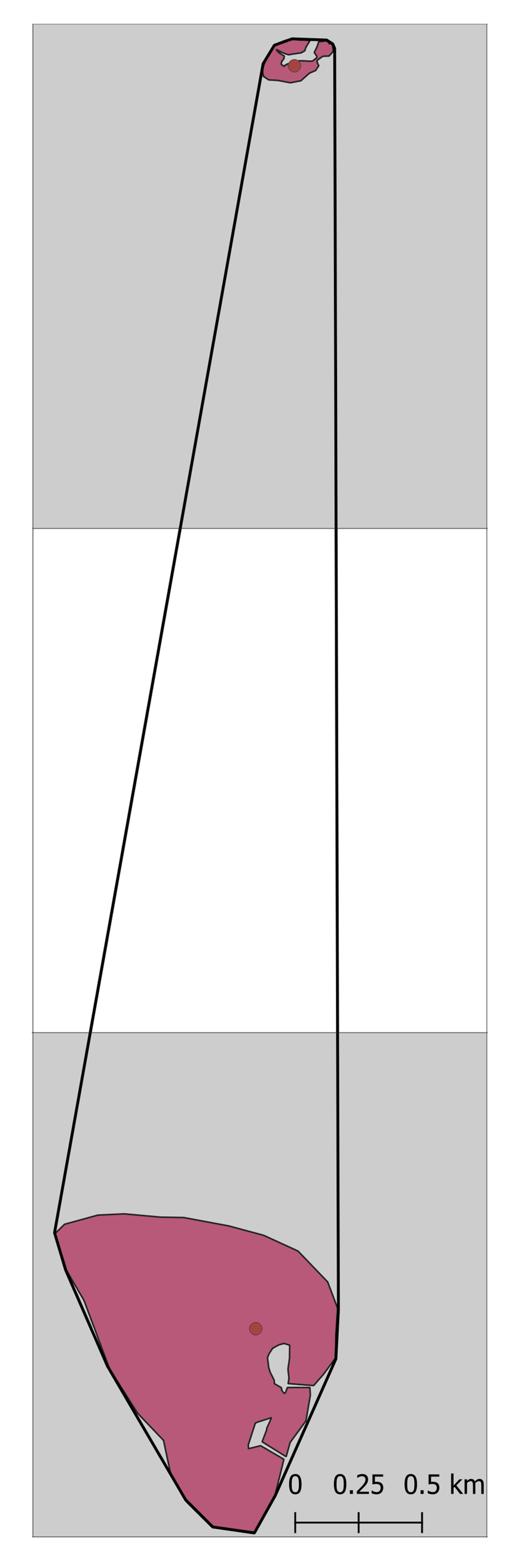

Supplement: S10 Fig — The black line represents the minimum convex polygon (MCP) of the extent of occurrence (EOO), pink polygons indicate mapped suitable habitat, red dots represent current records, and dark-shaded cells were accounted for in the estimation. All layers were created by the authors. Distribution was generated using field observations, habitat mapping, and altitude. No copyrighted or third-party material was used for the figure. (TIF) [file pone.0334746.s013.tif]

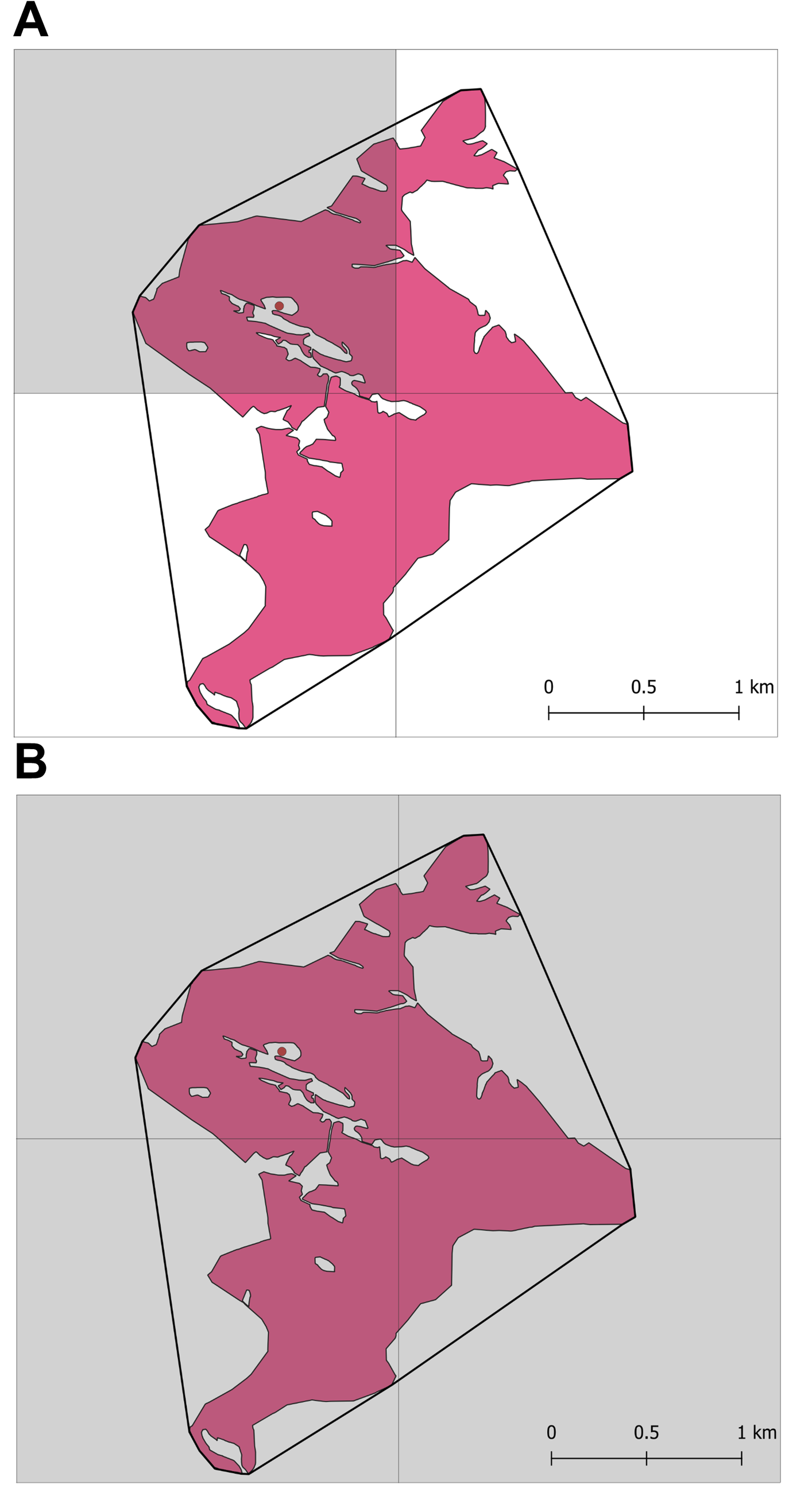

Supplement: S11 Fig — (A) Distribution considering the lower bound of area of occupancy (AOO) based on current records. (B) Distribution considering the upper bound of AOO incorporating suitable habitat. The black line represents the minimum convex polygon (MCP) of the extent of occurrence (EOO), pink polygons indicate mapped suitable habitat, the red dot represents the current record, and dark-shaded cells were accounted for in the estimation. All layers were created by the authors. Distribution was generated using field observations, habitat mapping, and altitude. No copyrighted or third-party material was used for the figure. (TIF) [file pone.0334746.s014.tif]

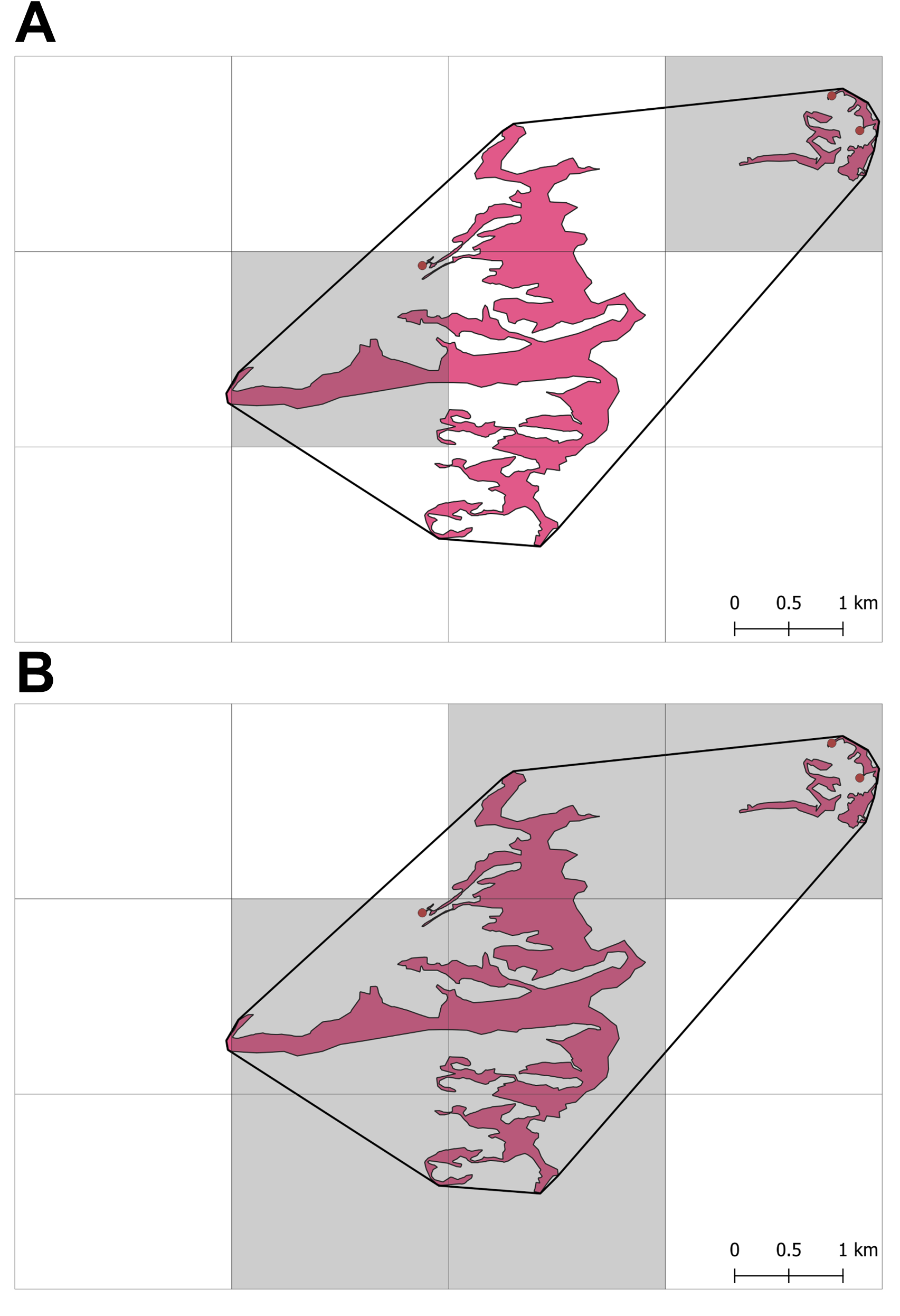

Supplement: S12 Fig — (A) Distribution considering the lower bound of area of occupancy (AOO) based on current records. (B) Distribution considering the upper bound of AOO incorporating suitable habitat. The black line represents the minimum convex polygon (MCP) of the extent of occurrence (EOO), pink polygons indicate mapped suitable habitat, red dots represent current record, and dark-shaded cells were accounted for in the estimation. All layers were created by the authors. Distribution was generated using field observations, habitat mapping, and altitude. No copyrighted or third-party material was used for the figure. (TIF) [file pone.0334746.s015.tif]

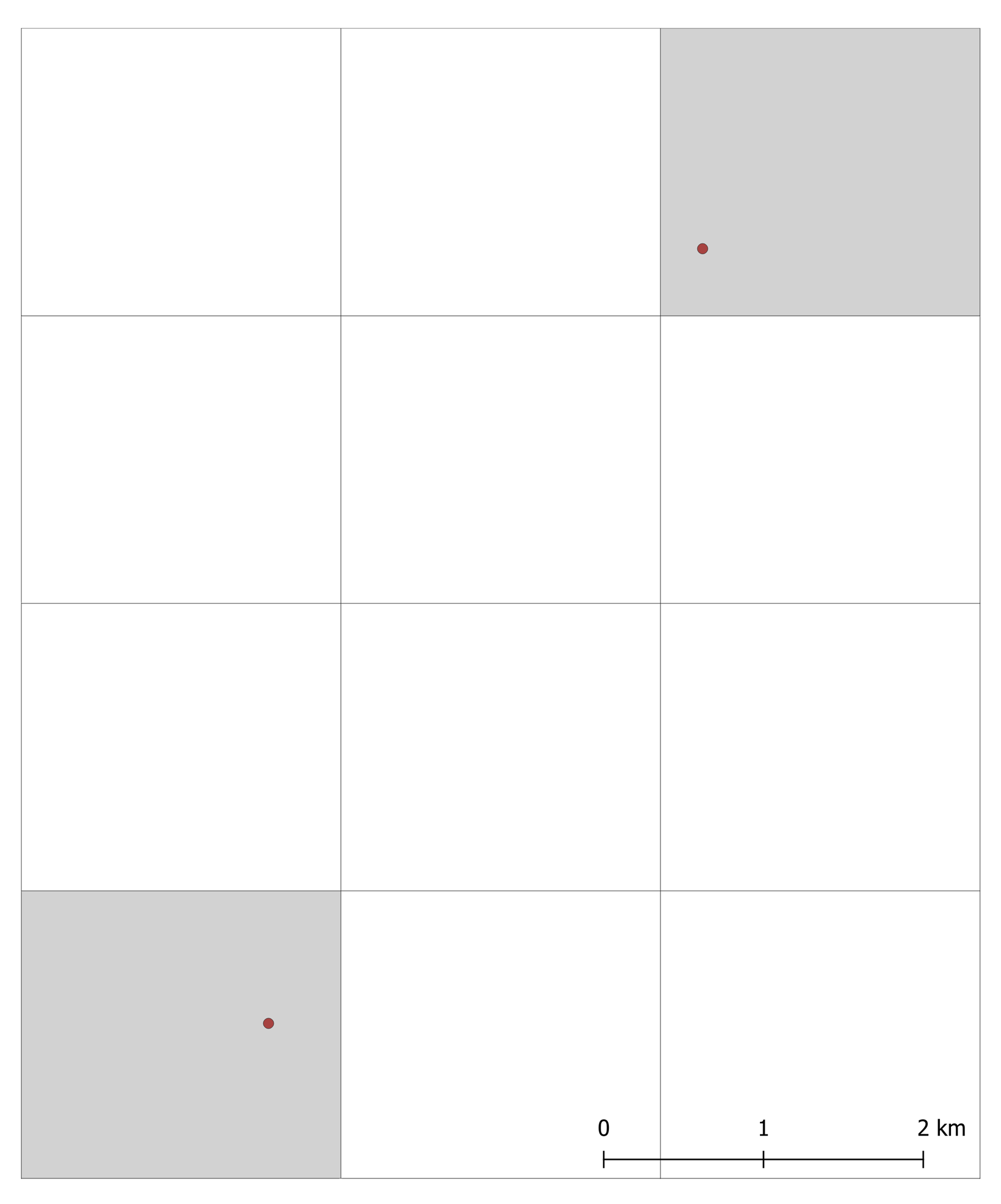

Supplement: S13 Fig — Red dots represent current records, and dark-shaded cells were accounted for in the estimation. All layers were created by the authors. Distribution was generated using field observations, habitat mapping, and altitude. No copyrighted or third-party material was used for the figure. (TIF) [file pone.0334746.s016.tif]

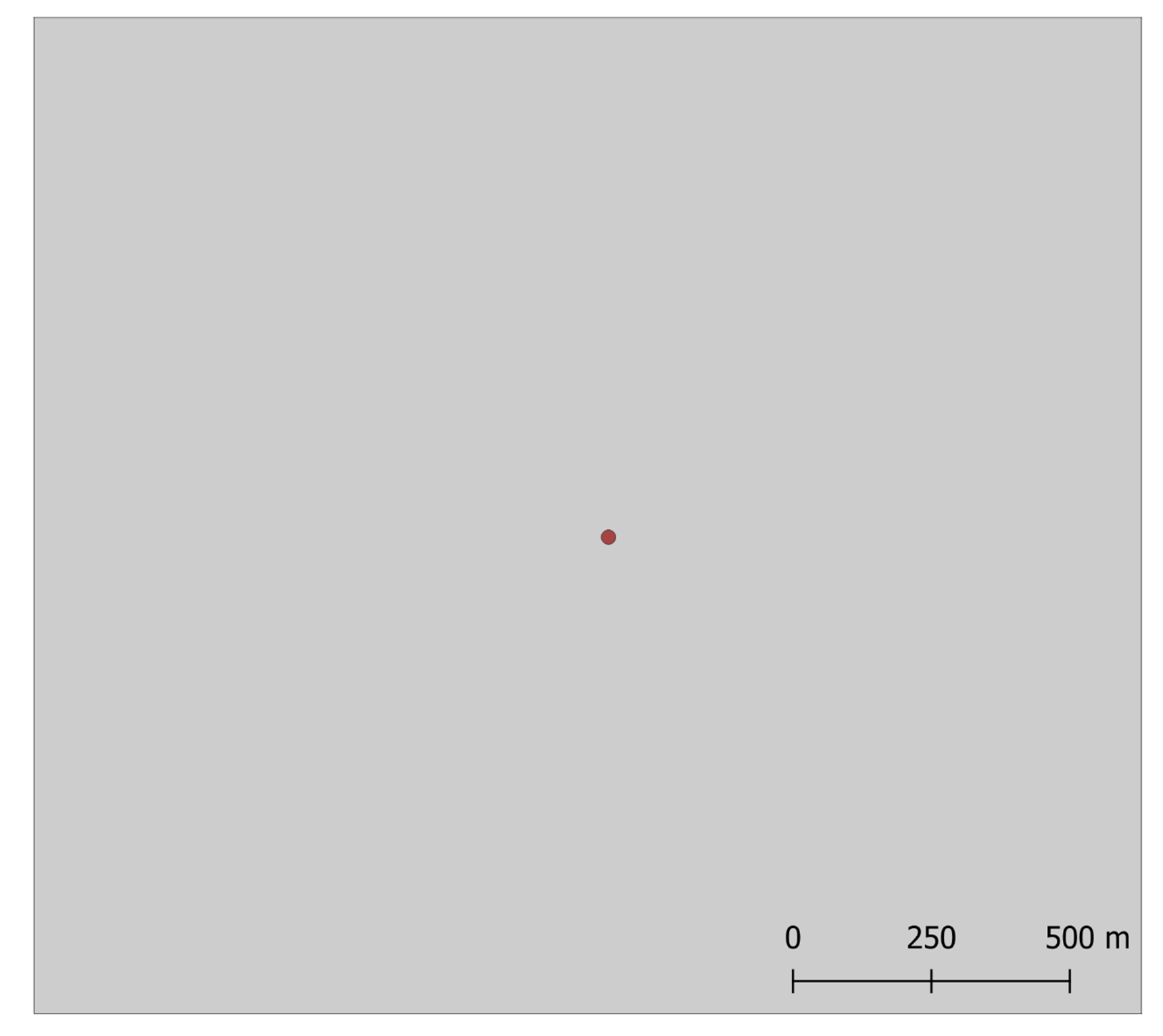

Supplement: S14 Fig — The red dot represents the current record, and the dark-shaded area was accounted for in the estimation. All layers were created by the authors. Distribution was generated using field observations, habitat mapping, and altitude. No copyrighted or third-party material was used for the figure. (TIF) [file pone.0334746.s017.tif]

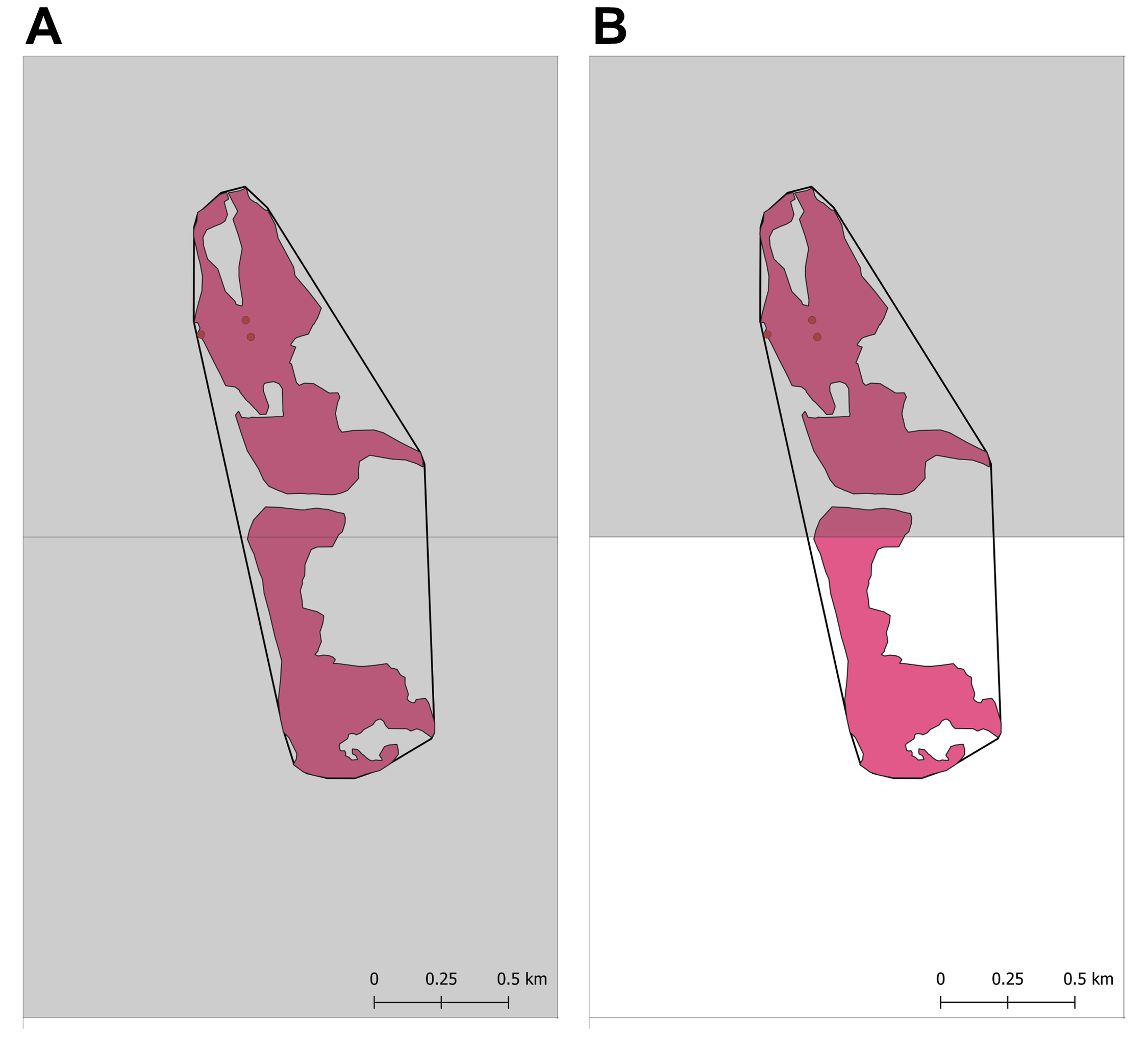

Supplement: S15 Fig — (A) Distribution considering the lower bound of area of occupancy (AOO) based on current records. (B) Distribution considering the upper bound of AOO incorporating suitable habitat. The black line represents the minimum convex polygon (MCP) of the extent of occurrence (EOO), pink polygons indicate mapped suitable habitat, red dots represent current records, and dark-shaded cells were accounted for in the estimation. All layers were created by the authors. Distribution was generated using field observations, habitat mapping, and altitude. No copyrighted or third-party material was used for the figure. (TIF) [file pone.0334746.s018.tif]

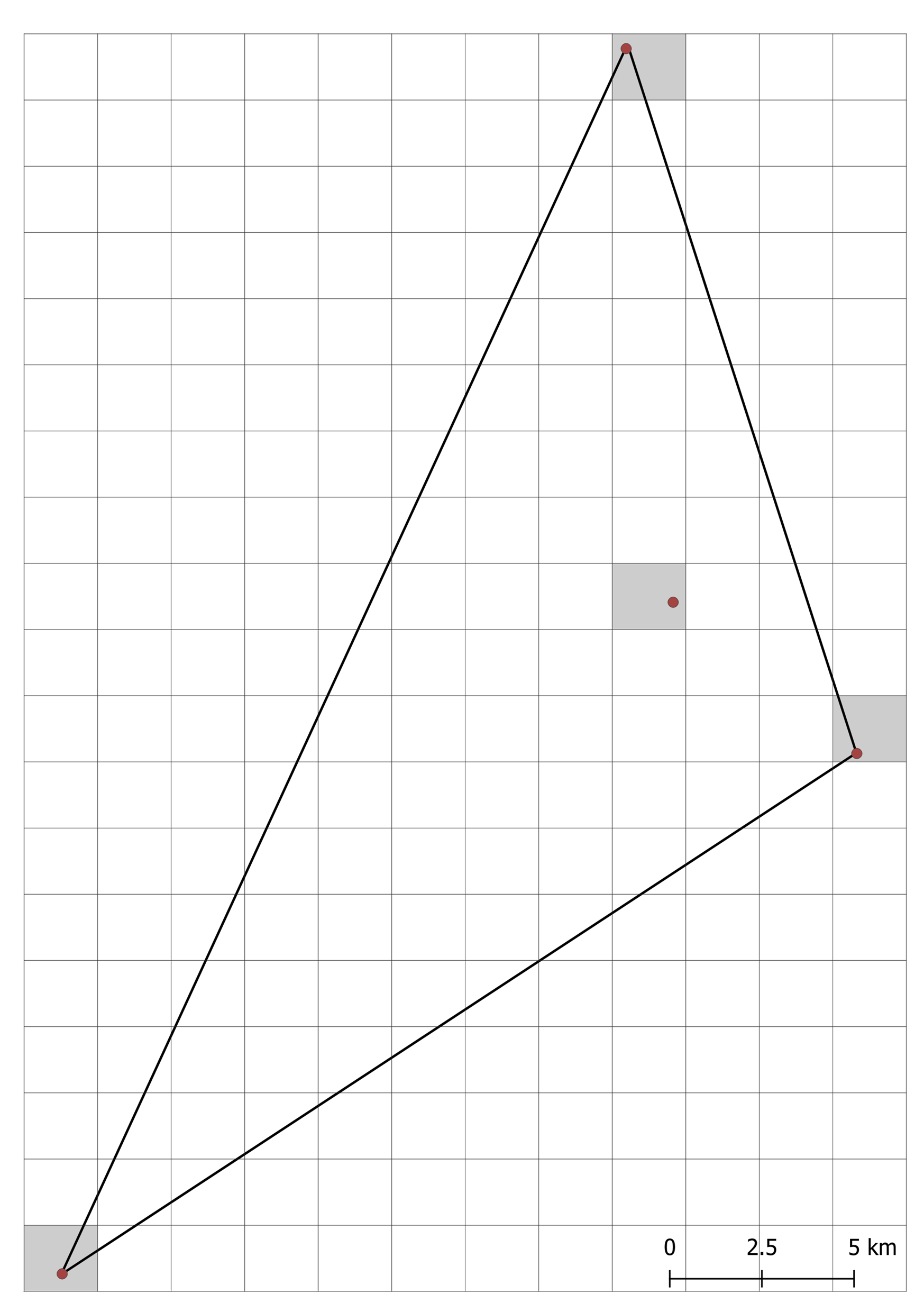

Supplement: S16 Fig — The black line represents the minimum convex polygon (MCP) of the extent of occurrence (EOO), dots represent current records, and dark-shaded cells were accounted for in the estimation. All layers were created by the authors. Distribution was generated using field observations, habitat mapping, and altitude. No copyrighted or third-party material was used for the figure. (TIF) [file pone.0334746.s019.tif]

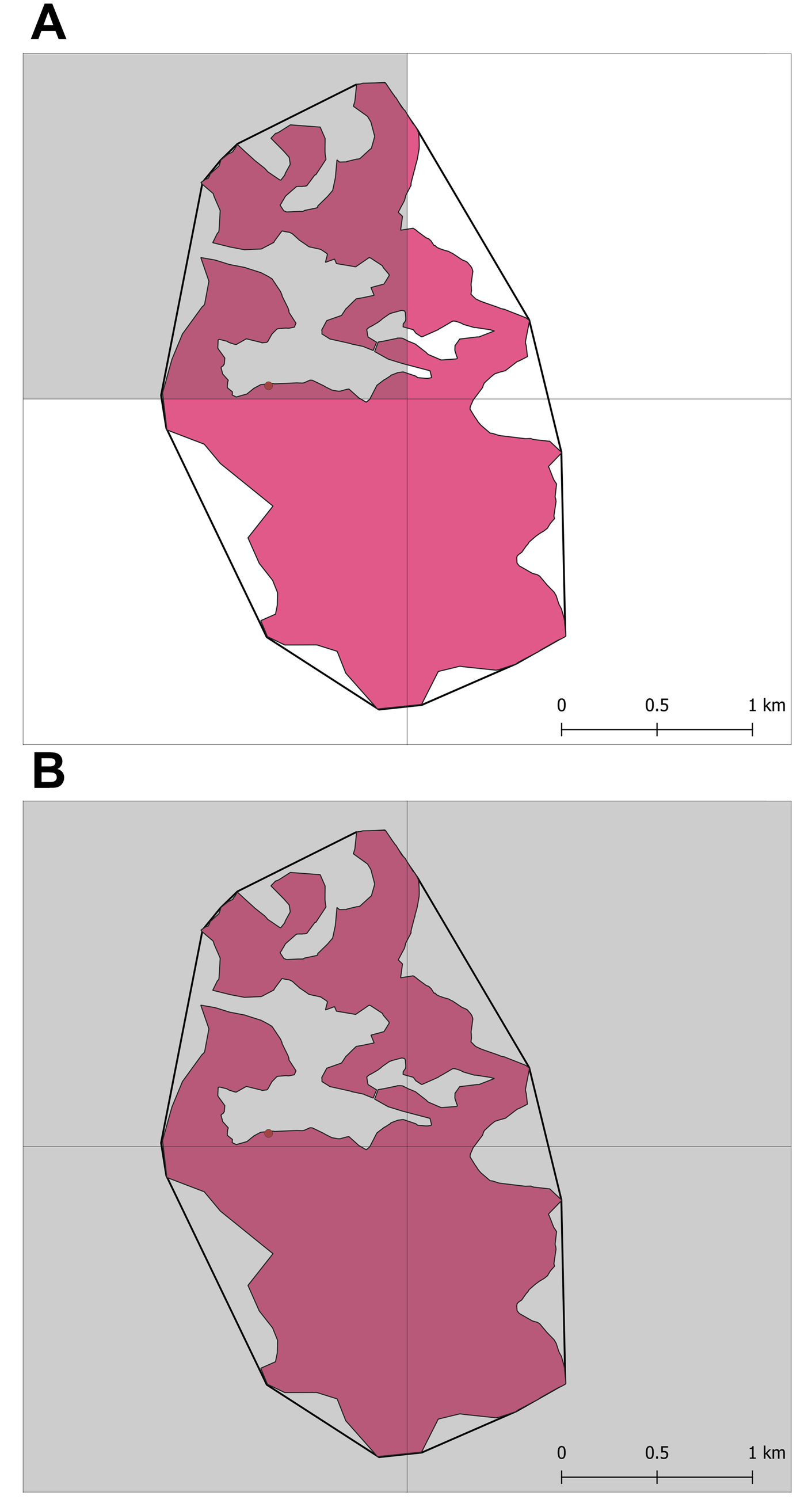

Supplement: S17 Fig — (A) Distribution considering the lower bound of area of occupancy (AOO) based on current records. (B) Distribution considering the upper bound of AOO incorporating suitable habitat. The black line represents the minimum convex polygon (MCP) of the extent of occurrence (EOO), pink polygons indicate mapped suitable habitat, the red dot represents the current record, and dark-shaded cells were accounted for in the estimation. All layers were created by the authors. Distribution was generated using field observations, habitat mapping, and altitude. No copyrighted or third-party material was used for the figure. (TIF) [file pone.0334746.s020.tif]

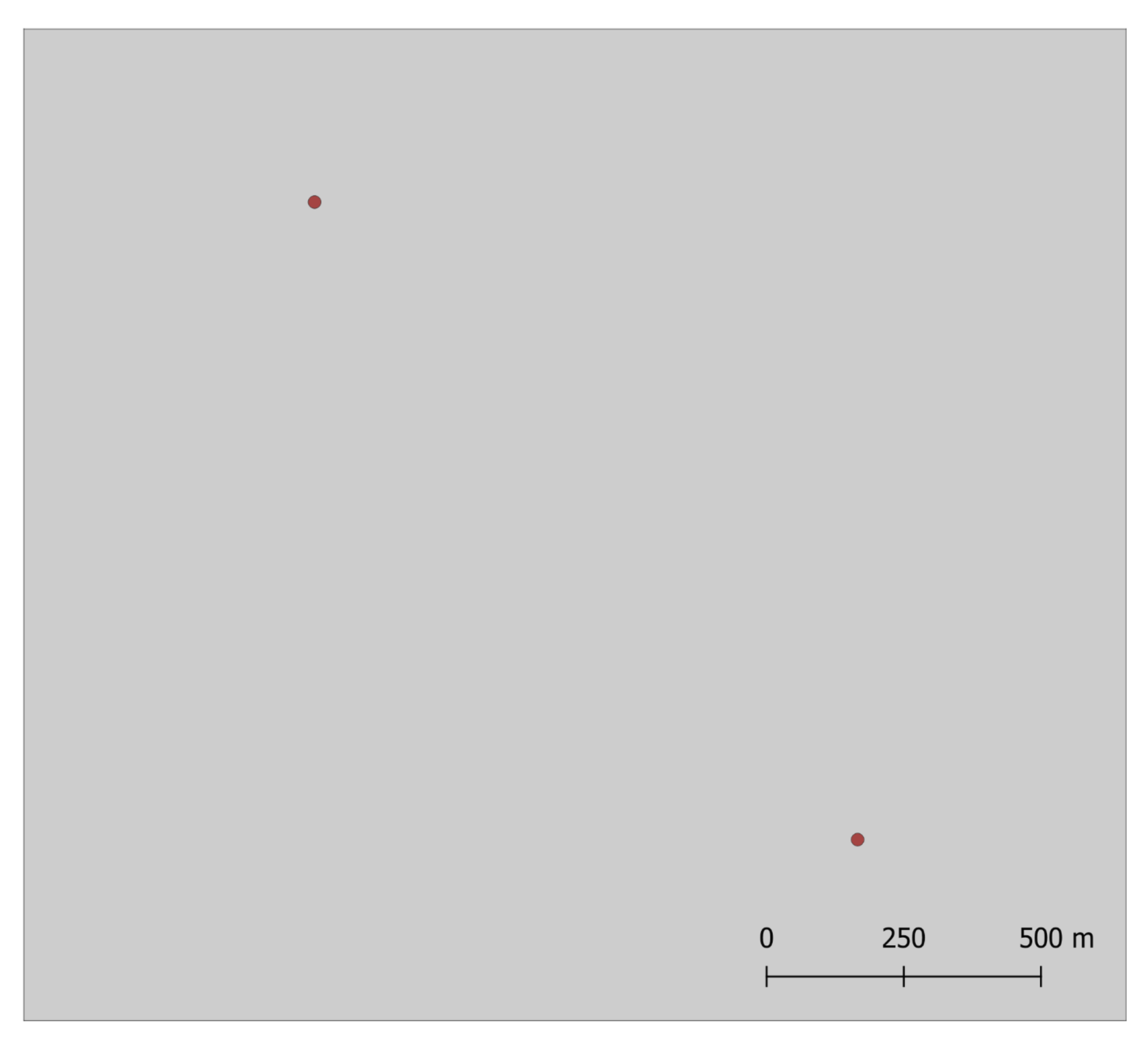

Supplement: S18 Fig — Red dots represent current records, and the dark-shaded area was accounted for in the estimation. All layers were created by the authors. Distribution was generated using field observations, habitat mapping, and altitude. No copyrighted or third-party material was used for the figure. (TIF) [file pone.0334746.s021.tif]

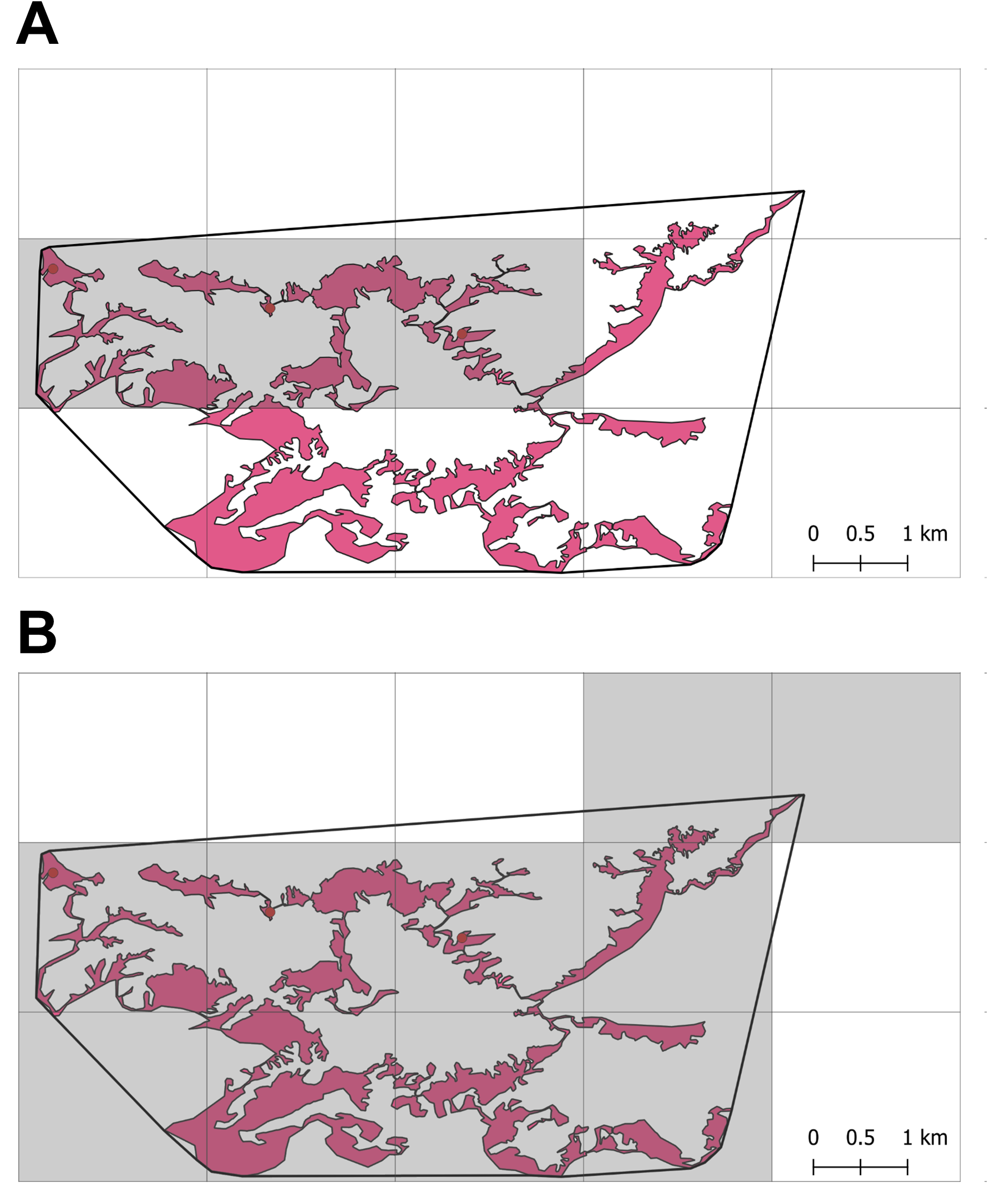

Supplement: S19 Fig — (A) Distribution considering the lower bound of area of occupancy (AOO) based on current records. (B) Distribution considering the upper bound of AOO incorporating suitable habitat. The black line represents the minimum convex polygon (MCP) of the extent of occurrence (EOO), pink polygons indicate mapped suitable habitat, red dots represent current records, and dark-shaded cells were accounted for in the estimation. All layers were created by the authors. Distribution was generated using field observations, habitat mapping, and altitude. No copyrighted or third-party material was used for the figure. (TIF) [file pone.0334746.s022.tif]

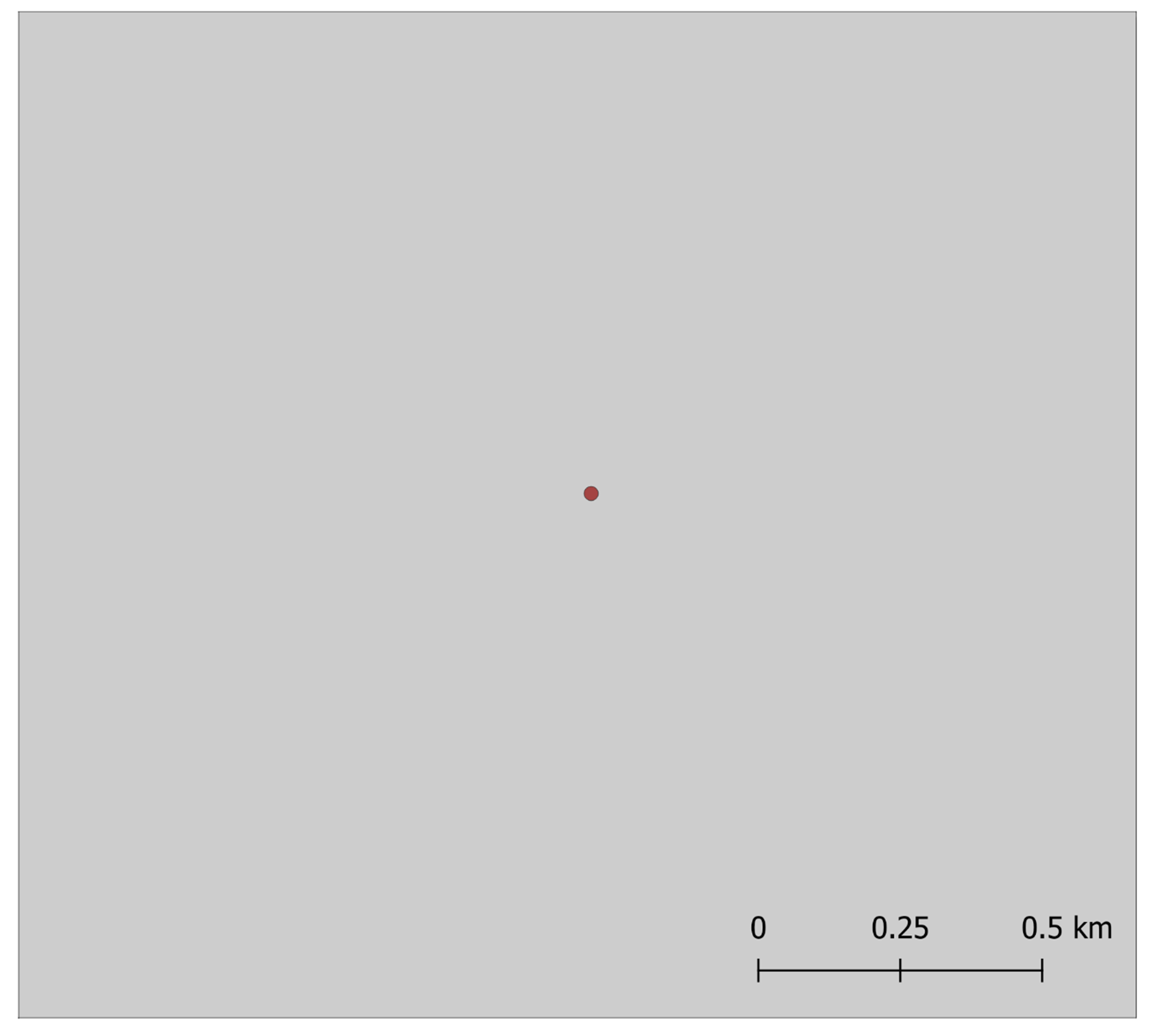

Supplement: S20 Fig — The red dot represents the current record, and the dark-shaded area was accounted for in the estimation. All layers were created by the authors. Distribution was generated using field observations, habitat mapping, and altitude. No copyrighted or third-party material was used for the figure. (TIF) [file pone.0334746.s023.tif]

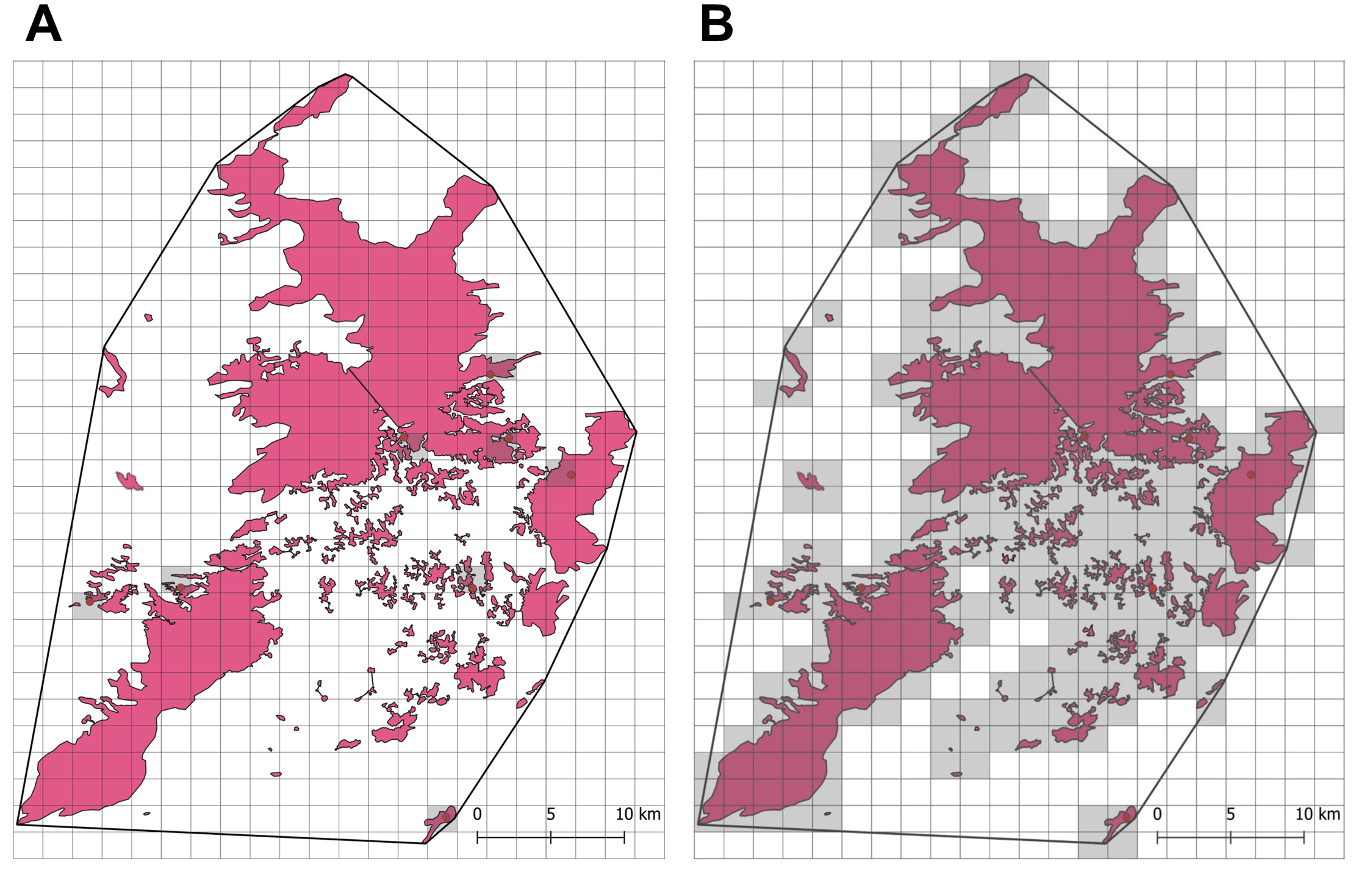

Supplement: S21 Fig — (A) Distribution considering the lower bound of area of occupancy (AOO) based on current records. (B) Distribution considering the upper bound of AOO incorporating suitable habitat. The black line represents the minimum convex polygon (MCP) of the extent of occurrence (EOO), pink polygons indicate mapped suitable habitat, red dots represent current records, and dark-shaded cells were accounted for in the estimation. All layers were created by the authors. Distribution was generated using field observations, habitat mapping, and altitude. No copyrighted or third-party material was used for the figure. (TIF) [file pone.0334746.s024.tif]

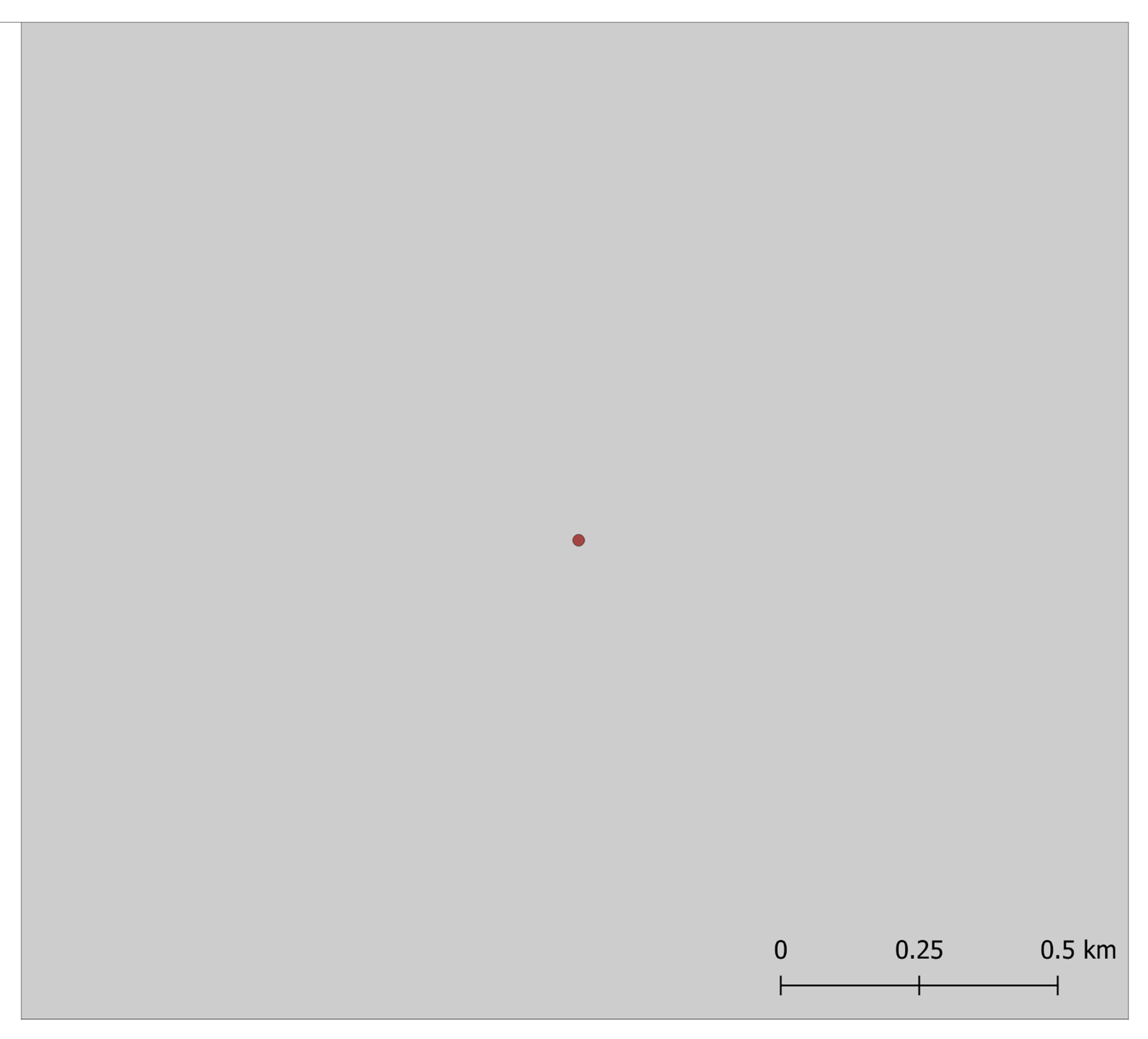

Supplement: S22 Fig — The red dot represents the current record, and the dark-shaded area was accounted for in the estimation. All layers were created by the authors. Distribution was generated using field observations, habitat mapping, and altitude. No copyrighted or third-party material was used for the figure. (TIF) [file pone.0334746.s025.tif]
