# Supplementary material for: A new species of Brachycephalus (Anura: Brachycephalidae) from Serra do Quiriri, northeastern Santa Catarina state, southern Brazil, with a review of the diagnosis among species of the B. pernix group and proposed conservation measures
Source: PLoS One. 2025 Dec 10;20(12):e0334746. doi: 10.1371/journal.pone.0334746 (PMC12694819; doi:10.1371/journal.pone.0334746)
Supplement: S1 Appendix — Abbreviations: CFBH = Célio F. B. Haddad collection, Departamento de Zoologia, Universidade Estadual Paulista, Campus de Rio Claro, São Paulo; DZUP = Coleção Herpetológica do Departamento de Zoologia, Universidade Federal do Paraná, Curitiba, Paraná; MHNCI = Museu de História Natural Capão da Imbuia, Curitiba, Paraná; MNRJ = Museu Nacional, Rio de Janeiro, Rio de Janeiro; MZUSP = Museu de Zoologia da Universidade de São Paulo, São Paulo, São Paulo; and ZUEC = Museu de História Natural, Universidade Estadual de Campinas, Campinas, São Paulo. (DOCX) [file pone.0334746.s026.docx]

**Appendix 1**

Examined specimens of *Brachycephalus*. Abbreviations: CFBH = Célio F. B. Haddad collection, Departamento de Zoologia, Universidade Estadual Paulista, Campus de Rio Claro, São Paulo; DZUP = Coleção Herpetológica do Departamento de Zoologia, Universidade Federal do Paraná, Curitiba, Paraná; MHNCI = Museu de História Natural Capão da Imbuia, Curitiba, Paraná; MNRJ = Museu Nacional, Rio de Janeiro, Rio de Janeiro; MZUSP = Museu de Zoologia da Universidade de São Paulo, São Paulo, São Paulo; and ZUEC = Museu de História Natural, Universidade Estadual de Campinas, Campinas, São Paulo.

***Brachycephalus actaeus*** (N = 12). SANTA CATARINA: Forte Marechal Luz, Ilha de São Francisco, municipality of São Francisco do Sul MHNCI 11624–5; Serra da Tiririca, municipality of Itapopá MHNCI 10832–4, 11024–30.

***Brachycephalus albolineatus*** (N = 32). SANTA CATARINA: Morro Azul, on the border between the municipalities of Pomerode and Rio dos Cedros MHNCI 10291, 10301, 10846, MHNCI eight uncatalogued specimens; Morro Boa Vista, boundary of the municipalities of Jaraguá do Sul and Massaranduba MHNCI 10290 (holotype), MHNCI 10295–300 (paratypes), MNRJ 90349 (paratype), MHNCI 10293 (juvenile), 11563–5 (all topotypes); Morro do Garrafão, municipality of Corupá MHNCI 10836–42; Morro do Schmidt, municipality of Pomerode MHNCI two uncatalogued specimens.

***Brachycephalus alipioi*** (N = 9). ESPÍRITO SANTO: Alto Castelinho, municipality of Vargem Alta MHNCI 10804–6, 10844, MHNCI five uncatalogued specimens.

***Brachycephalus auroguttatus*** (N = 22). SANTA CATARINA: Pedra da Tartaruga, municipality of Garuva MHNCI 10200 (holotype), MHNCI 10198–9, 10201–9, 10211–4 (all paratypes); Trail to Pedra da Tartaruga, municipality of Garuva MHNCI 10849, 11768, and four uncatalogued specimens.

***Brachycephalus boticario*** (N = 18). SANTA CATARINA: Morro do Cachorro, boundary of the municipalities of Blumenau, Gaspar, and Luiz Alves MHNCI 10257 (holotype), MHNCI 10253–6, 10258–9, 10731 (all paratypes), MHNCI 10253–9 (topotypes), MHNCI 11557–9 (topotypes).

***Brachycephalus brunneus*** (N = 52). PARANÁ: Abrigo 1, municipality of Campina Grande do Sul MHNCI three uncatalogued specimens; Camapuã, Serra dos Órgãos, boundary of the municipalities of Campina Grande do Sul and Antonina MHNCI 10165–74, 10730–2, 10787, MHNCI 14 uncatalogued specimens; Caratuva, Serra dos Órgãos, municipality of Campina Grande do Sul MNRJ 40289–91 (paratypes), MHNCI 1919–20, 10175–84, 10729, 11551–2 (all topotypes); Mãe Catira, Serra da Graciosa, municipality of Quatro Barras MHNCI 10784, MHNCI two uncatalogued specimens.

***Brachycephalus coloratus*** (N = 20). PARANÁ: Estância Hidroclimática Recreio da Serra, Serra da Baitaca, municipality of Piraquara MHNCI 10273 (holotype), MHNCI 10274–9 (paratypes), MNRJ 89949–50 (paratypes), MHNCI 11566–8, 11593–5, 11626–9 (all topotypes); Pão de Ló, Serra da Baitaca, municipality of Quatro Barras MHNCI 11596.

***Brachycephalus curupira*** (N = 15). PARANÁ: Morro do Canal, municipality of Piraquara MHNCI 10724–8; Morro do Vigia, municipality of Piraquara MHNCI 10723; Serra do Salto, Malhada District, municipality of São José dos Pinhais MHNCI 10280 (holotype), MHNCI 10281–7, 10292 (all paratypes).

***Brachycephalus didactylus*** (N = 16). RIO DE JANEIRO: Municipality of Engenheiro Paulo de Frontin ZUEC 10825; Rodovia Santos Dumont, near to Mirante do Soberbo, municipality of Teresópolis MHNCI 11615; Sacra Família do Tinguá, municipality of Engenheiro Paulo de Frontin ZUEC 1132–3 (topotypes), MZUSP 13613–20, 64810–1, 94621 (topotypes), MHNCI 11614 (topotype).

***Brachycephalus ephippium*** (N = 9). RIO DE JANEIRO: Parque Nacional Serra dos Órgãos MZUSP 104140–7; Vale de Revolta MCZ A–108655.

***Brachycephalus ferruginus*** (N = 9). PARANÁ: Olimpo, Serra do Marumbi, municipality of Morretes MHNCI 125, 128, 10230, 11630–4 (all topotypes), MHNCI one uncatalogued specimen (topotype).

***Brachycephalus fuscolineatus*** (N = 13). SANTA CATARINA: Morro Braço da Onça, municipality of Luiz Alves MHNCI 10850–1; Morro do Baú, municipality of Ilhota MHNCI 10231 (holotype), 10230 (juvenile paratype), MHNCI 10232–7 (paratypes), MHNCI three uncatalogued (topotypes).

***Brachycephalus hermogenesi*** (N = 15). SÃO PAULO: Corcovado, municipality of Ubatuba MHNCI 10823–5; Núcleo Cunha, Parque Estadual da Serra do Mar, municipality of Cunha MHNCI 11592; Picinguaba, Parque Estadual da Serra do Mar, municipality of Ubatuba ZUEC 9715 (holotype), ZUEC 9716–25 (paratypes).

***Brachycephalus izecksohni*** (N = 7). PARANÁ: Torre da Prata, Serra da Prata, boundary of the municipalities of Morretes, Paranaguá, and Guaratuba CFBH 7381–2, 7384 (all paratypes), MHNCI 10835, MHNCI three uncatalogued specimens (all topotypes).

***Brachycephalus leopardus*** (N = 41) PARANÁ: Morro dos Perdidos, municipality of Guaratuba MHNCI 10783, MHNCI nine uncatalogued specimens; Serra do Araçatuba, municipality of Tijucas do Sul MHNCI 10250 (holotype), MHNCI 10239–49, 10251, 10252 (juvenile) (all paratypes), MHNCI 11620–3 (topotypes), MHNCI 13 uncatalogued specimens (topotypes).

***Brachycephalus mariaeterezae*** (N = 13). SANTA CATARINA: Reserva Particular do Patrimônio Natural Caetezal, top of the Serra Queimada, municipality of Joinville MHNCI 9811 (holotype), MHNCI 9812 and 10190–7 (paratypes).

***Brachycephalus mirissimus*** (N = 14). SANTA CATARINA: Morro Santo Anjo, municipality of Massaranduba MHNCI 10793 (holotype), MHNCI 10794–803 (paratypes), MHNCI 11560–2 (topotypes).

***Brachycephalus nodoterga*** (N = 6). SÃO PAULO: Reserva Biológica Tamboré, municipality of Santana de Parnaíba MZUSP 147711–6.

***Brachycephalus olivaceus*** (N = 20)*.* SANTA CATARINA: Base of the Serra Queimada, municipality of Joinville MHNCI 9813 (holotype), MHNCI 10238, 11605–9 (all paratypes); Castelo dos Bugres, municipality of Joinville MHNCI 9814–8 (paratypes), MHNCI 11600–4; Morro do Boi, municipality of Corupá MHNCI 10288–9; Pico Jurapê, municipality of Joinville MHNCI 11610.

***Brachycephalus pernix*** (N = 33). PARANÁ: Anhangava, Serra da Baitaca, municipality of Quatro Barras MNRJ 17349 (holotype), CFBH 2597–8 (paratypes), MHNCI 1818–9, 3000–4 (all paratypes), ZUEC 9433–7 (paratypes), MHNCI 1820, 9806–10, 10153–64 (all topotypes).

***Brachycephalus pitanga*** (N = 6). SÃO PAULO: Trilha do Ipiranga 50 m from the Rio Ipiranga, Núcleo Santa Virgínia, Parque Estadual da Serra do Mar, municipality of São Luiz do Paraitinga MHNCI 10733–4, 10821–2, 10843 (all topotypes), MHNCI one uncatalogued specimen (topotype).

***Brachycephalus pombali*** (N = 39)*.* PARANÁ: Morro dos Padres, Serra da Igreja, municipality of Morretes CFBH 8042 (holotype), 8043–53 (paratypes), MHNCI 11553–6, 11569–70, 11611–5, 11618–9 (all topotypes), MHNCI 14 uncatalogued specimens (topotypes).

***Brachycephalus quiririensis*** (N = 21). SANTA CATARINA: Serra do Quiriri, municipality of Campo Alegre MHNCI 10261 (holotype), MHNCI 10260, 10262–72 (all paratypes), MHNCI eight uncatalogued specimens (topotypes).

***Brachycephalus sulfuratus*** (N = 37). SÃO PAULO: Bairro Rio Vermelho, municipality of Barra do Turvo MHNCI 11558; Base of the Serra Água Limpa, municipality of Apiaí MHNCI 11583; Near Jurupará dam, municipality of Piedade MHNCI 10790–2, 10829–31; Torre Embratel, Parque Estadual do Rio Turvo, municipality of Cajati MHNCI 11588–9. PARANÁ: Caratuval, near the Parque Estadual das Lauráceas, municipality of Adrianópolis MHNCI 11571; Corvo, municipality of Quatro Barras MHNCI 10303, 11572–8, 10788; Entroncamento Teba, Rio Turvo, municipality of Campina Grande do Sul MHNCI 11556–7; Estância Hidroclimática Recreio da Serra, Serra da Baitaca, municipality of Piraquara MHNCI 11591; Fazenda Thalia, municipality of Balsa Nova MHNCI 11579–82; Mananciais da Serra, municipality of Piraquara MHNCI 10302; Olimpo, Serra do Marumbi, municipality of Morretes MHNCI one uncatalogued specimen; Parque Estadual das Lauráceas, next to the park headquarters, municipality of Adrianópolis MHNCI 11590; Pedra Branca do Araraquara, Serra do Araraquara, municipality of Guaratuba DZUP 400; Recanto das Hortências, municipality of São José dos Pinhais MHNCI 11591; Salto do Inferno, Rio Capivari, municipality of Bocaiúva do Sul MHNCI 9800. SANTA CATARINA: Morro do Garrafão, municipality of Corupá MHNCI 10826–8; Pedra da Tartaruga, municipality of Garuva MHNCI 11585.

***Brachycephalus tabuleiro*** (N = 4). SANTA CATARINA: Afluente da margem direita do rio do Ponche, Serra do Tabuleiro, municipality of São Bonifácio MHNCI 11498–9 (paratypes), MHNCI 12380–1 (topotypes).

***Brachycephalus toby*** (N = 3). SÃO PAULO: Morro do Corcovado, Parque Estadual da Serra do Mar, municipality of Ubatuba MHNCI 10807–9 (topotypes).

***Brachycephalus tridactylus*** (N = 51). SÃO PAULO: Bairro Rio Vermelho, municipality of Barra do Turvo MHNCI 11643–5; Estrada das Conchas, municipality of Barra do Turvo MHNCI 11642; Fazenda Fronteira, municipality of Barra do Turvo MHNCI 11648–50, MHNCI three uncatalogued specimens; Morro do Bisel, Serra do Guaraú, municipality of Cajati MHNCI 11637; Serra do Pinheiro, municipality of Cajati MHNCI 11638–41; Serra Pelada, municipality of Barra do Turvo MHNCI 11571, 11646–7, MHNCI one uncatalogued specimen; Torre Embratel, municipality of Cajati MHNCI 10848, 10852, 11630–6, MHNCI 11 uncatalogued specimens. PARANÁ: Serra do Morato, Reserva Natural Salto Morato, municipality of Guaraqueçaba MHNCI 10185–9, 10294, 10729–30 (all topotypes), CFBH 43887–90 (topotypes).

***Brachycephalus verrucosus*** (N = 30). SANTA CATARINA: Morro da Tromba, municipality of Joinville MHNCI 9819 (holotype), MHNCI 9820, 10215–29 (all paratypes), MHNCI 13 uncatalogued specimens (topotypes).

***Brachycephalus vertebralis*** (N = 11). RIO DE JANEIRO/SÃO PAULO: Morro Cuscuzeiro, Núcleo Picinguaba of the Parque Estadual da Serra do Mar and Parque Nacional da Serra da Bocaina, boundary of the municipalities of Parati, Rio de Janeiro state, and Ubatuba, São Paulo state MHNCI 10810–20.

***Brachycephalus* sp.** (N = 59). SÃO PAULO: Municipality of Cotia MHNCI 2611–6. PARANÁ: Chapeuzinho, boundary of the municipalities of Morretes and Piraquara DZUP 502–4; Serra Canasvieiras, boundary of the municipalities of Guaratuba and Morretes MHNCI 10785, DZUP 452–3; Tupipiá, Serra dos Órgãos, municipality of Antonina MHNCI 10786, 10789, 10845, DZUP 507–13, 515–6, 601–2. SANTA CATARINA: Morro da Pedra, municipality of Navegantes MHNCI two uncatalogued specimens; Morro da Prata, municipality of Ilhota MHNCI four uncatalogued specimens; Morro dos Monos; municipality of Luiz Alves MHNCI two uncatalogued specimens; Pedra Branca do Araraquara, Serra do Araraquara, municipality of Guaratuba MHNCI one uncatalogued specimen; Serra do Pico, municipality of Joinville MHNCI 24 uncatalogued specimens.
