# Supplementary material for: A new species of Brachycephalus (Anura: Brachycephalidae) from Serra do Quiriri, northeastern Santa Catarina state, southern Brazil, with a review of the diagnosis among species of the B. pernix group and proposed conservation measures
Source: PLoS One. 2025 Dec 10;20(12):e0334746. doi: 10.1371/journal.pone.0334746 (PMC12694819; doi:10.1371/journal.pone.0334746)
Supplement: S2 Appendix — Sample size indicates number of analyzed calls and individuals (some recordings can contain more than one call). Abbreviation: MHNCI = Museu de História Natural Capão da Imbuia, Curitiba, Paraná; CASA = Coleção Audiovisual do Semiárido, Mossoró, Rio Grande do Norte; FNJV = Fonoteca Neotropical Jacques Vielliard, Campinas, São Paulo. Recordings at MHNCI were made by the authors with the following devices: digital recorder Sony PCM-D50 with a Sennheiser ME 66/K6 microphone, digital recorder Marantz PMD660 with a Sennheiser ME 66/K6 microphone, and/or digital recorder Tascam DR-44WL with a Sennheiser ME 67/K6 microphone, with sampling frequency rate of 44.1 kHz and 16-bit resolution. (DOCX) [file pone.0334746.s027.docx]

**Appendix 2**

Advertisement calls and territorial calls of *Brachycephalus* analyzed. Sample size indicates number of analyzed calls and individuals (some recordings can contain more than one call). Abbreviation: MHNCI = Museu de História Natural Capão da Imbuia, Curitiba, Paraná; CASA = Coleção Audiovisual do Semiárido, Mossoró, Rio Grande do Norte; FNJV = Fonoteca Neotropical Jacques Vielliard, Campinas, São Paulo. Recordings at MHNCI were made by the authors with the following devices: digital recorder Sony PCM-D50 with a Sennheiser ME 66/K6 microphone, digital recorder Marantz PMD660 with a Sennheiser ME 66/K6 microphone, and/or digital recorder Tascam DR-44WL with a Sennheiser ME 67/K6 microphone, with sampling frequency rate of 44.1 kHz and 16-bit resolution.

**Advertisement calls**

***Brachycephalus actaeus*** (N = 21/15). SANTA CATARINA: Forte Marechal Luz, Ilha de São Francisco, municipality of São Francisco do Sul MHNCI 365–9; Serra da Palha, Laranjeiras, Ilha de São Francisco, municipality of São Francisco do Sul MHNCI 289; Serra da Tiririca, municipality of Itapoá MHNCI 280–8, MHNCI 302–7.

***Brachycephalus albolineatus*** (N = 34/20). SANTA CATARINA: Morro Boa Vista, boundary of the municipalities of Jaraguá do Sul and Massaranduba MHNCI 001–34.

***Brachycephalus auroguttatus*** (N = 8/6). SANTA CATARINA: Trail to Pedra da Tartaruga, municipality of Garuva MHNCI 381–8.

***Brachycephalus boticario*** (N = 16/8). SANTA CATARINA: Morro do Cachorro, boundary of the municipalities of Blumenau, Gaspar, and Luiz Alves MHNCI 138–46, MHNCI 148, MHNCI 160.

***Brachycephalus brunneus*** (N = 19/20). PARANÁ: Caratuva, Serra dos Órgãos, municipality of Campina Grande do Sul MHNCI 083–96; Caranguejeira, Serra da Graciosa, municipality of Quatro MHNCI 349–53.

***Brachycephalus coloratus*** (N = 9/5). PARANÁ: Estância Hidroclimática Recreio da Serra, Serra da Baitaca, municipality of Piraquara MHNCI 245–50, MHNCI 354–6.

***Brachycephalus curupira*** (N = 26/26). PARANÁ: Morro do Canal, municipality of Piraquara MHNCI 097–106; Serra do Salto, Malhada District, municipality of São José dos Pinhais MHNCI 107–122.

***Brachycephalus didactylus*** (N = 100/9). RIO DE JANEIRO: Trail to Pedra do Elefante, municipality of Teresópolis MHNCI 389–97.

***Brachycephalus ferruginus*** (N = 12/7). PARANÁ: Olimpo, Serra do Marumbi, municipality of Morretes MHNCI 290–301.

***Brachycephalus fuscolineatus*** (N = 11/7). SANTA CATARINA: Morro Braço da Onça, municipality of Luiz Alves MHNCI 345–6, MHNCI 357–63; Morro do Baú, municipality of Ilhota, Santa Catarina MHNCI 370–1.

***Brachycephalus hermogenesi*** (N = 34/25). SÃO PAULO: Corcovado, municipality of Ubatuba MHNCI 165; Estação Biológica de Boracéia, municipality of Salesópolis MHNCI 166–9; Morro do Cantagalo, municipality of Caraguatatuba MHNCI 222–3; Núcleo Cunha, Parque Estadual da Serra do Mar, municipality of Cunha MHNCI 170–1; Núcleo Picinguaba, Parque Estadual da Serra do Mar, municipality of Ubatuba MHNCI 172–87; Parque Natural Municipal Nascentes de Paranapiacaba, municipality of Santo André MHNCI 213–6; Trilha do Ipiranga 50 m from the Rio Ipiranga, Núcleo Santa Virgínia, Parque Estadual da Serra do Mar, municipality of São Luiz do Paraitinga MHNCI 188–92.

***Brachycephalus izecksohni*** (N = 3/1). PARANÁ: Torre da Prata, Serra da Prata, boundary of the municipalities of Morretes, Paranaguá, and Guaratuba MHNCI 256–8.

***Brachycephalus leopardus*** (N = 12/10). PARANÁ: Morro dos Perdidos, municipality of Guaratuba MHNCI 343–4, MHNCI 372–4; Serra do Araçatuba, municipality of Tijucas do Sul MHNCI 337–42, MHNCI 364.

***Brachycephalus lulai* sp. nov.** (N = 13/13). SANTA CATARINA: Monte Crista, municipality of Garuva MHNCI 233–6; Pico Garuva, municipality of Garuva MHNCI 224–32.

***Brachycephalus mirissimus*** (N = 31/12). SANTA CATARINA: Morro Santo Anjo, municipality of Massaranduba MHNCI 052–82.

***Brachycephalus olivaceus*** (N = 23/19)*.* SANTA CATARINA: Castelo dos Bugres, municipality of Joinville MHNCI 324–6; Morro do Boi, municipality of Corupá MHNCI 308–16; Pico Jurapê, municipality of Joinville MHNCI 317–23, MHNCI 375.

***Brachycephalus pernix*** (N = 8/8). PARANÁ: Anhangava, Serra da Baitaca, municipality of Quatro Barras MHNCI 251–5, MHNCI 376–8.

***Brachycephalus pombali*** (N = 8/8)*.* PARANÁ: Morro dos Padres, Serra da Igreja, municipality of Morretes MHNCI 259–64.

***Brachycephalus quiririensis*** (N = 12/9). SANTA CATARINA: Campos do Quiriri, Serra do Quiriri, on the border between the municipalities of Campo Alegre and Garuva FNJV 0040992; Bradador, Serra do Quiriri, municipality of Garuva MHNCI 329–32; Serra do Quiriri, municipality of Campo Alegre MHNCI 327–8, MHNCI 333–6.

***Brachycephalus sulfuratus*** (N = 175/23). SÃO PAULO: Base of the Serra Água Limpa, municipality of Apiaí MHNCI 129; Biquinha, municipality of Juquiá MHNCI 128; Near the Jurupará dam, municipality of Piedade MHNCI 123–5; Núcleo Itutinga–Pilões, Parque Estadual da Serra do Mar, municipality of Cubatão MHNCI 126–7; Serra do Guaraú, on the border of the municipalities of Cajati and Jacupiranga MHNCI 130; Torre Embratel, municipality of Cajati MHNCI 218. PARANÁ: Caratuval, near the Parque Estadual das Lauráceas, municipality of Adrianópolis MHNCI 131; Caratuval, Parque Estadual das Lauráceas, municipality of Adrianópolis MHNCI 132; Entroncamento Teba, Rio Turvo, municipality of Campina Grande do Sul MHNCI 219; Fazenda Thalia, municipality of Balsa Nova MHNCI 134; Morro do Canal, municipality of Piraquara MHNCI 220; Reserva Particular do Patrimônio Natural Salto Morato, municipality of Guaraqueçaba MHNCI 133. SANTA CATARINA: Monte Crista, municipality of Garuva MHNCI 221; Morro do Garrafão, municipality of Corupá MHNCI 137; Morro Garuva, municipality of Garuva MHNCI 136; Serra do Pico, municipality of Joinville MHNCI 217; Truticultura, municipality of Garuva MHNCI 135.

***Brachycephalus tabuleiro*** (N = 7/5). SANTA CATARINA: Afluente da margem direita do rio do Ponche, Serra do Tabuleiro, municipality of São Bonifácio CASA 154–7, MHNCI three uncatalogued recordings.

***Brachycephalus tridactylus*** (N = 17/15). PARANÁ: Serra do Morato, Reserva Natural Salto Morato, municipality of Guaraqueçaba MHNCI 035–51.

***Brachycephalus verrucosus*** (N = 11/11). SANTA CATARINA: Morro da Tromba, municipality of Joinville MHNCI 237–44, MHNCI 379–80.

***Brachycephalus* sp.** (N = 21/19). RIO DE JANEIRO: Trilha do Corisco, municipality of Paraty MHNCI 206–12. SÃO PAULO: Corcovado, municipality of Ubatuba MHNCI 193–205.

***Brachycephalus* sp.** (N = 11/10). PARANÁ: Tupipiá, Serra dos Órgãos, municipality of Antonina MHNCI 265–74.

***Brachycephalus* sp.** (N = 6/6). PARANÁ: Serra Canasvieiras, boundary of the municipalities of Guaratuba and Morretes MHNCI 275–9.

***Brachycephalus* sp.** (N = 2/2). SANTA CATARINA: Serra do Pico, municipality of Joinville MHNCI 347–8.

**Territorial calls**

***Brachycephalus didactylus*** (N = 28/3). RIO DE JANEIRO: Trail to Pedra do Elefante, municipality of Teresópolis MHNCI 397–8.
