# Supplementary material for: A new species of Brachycephalus (Anura: Brachycephalidae) from Serra do Quiriri, northeastern Santa Catarina state, southern Brazil, with a review of the diagnosis among species of the B. pernix group and proposed conservation measures
Source: PLoS One. 2025 Dec 10;20(12):e0334746. doi: 10.1371/journal.pone.0334746 (PMC12694819; doi:10.1371/journal.pone.0334746)
Supplement: Attachment — Submitted filename: PONE-D-24-43253_review.docx [file pone.0334746.s029.docx]

This manuscript proposes a new species of the anuran genus Brachycephalus. The authors present several lines of evidence, including external morphology, osteology, vocalizations and molecular data to support the status of this taxon as a new species. They also acknowledge the difficulty in differentiating many species of the B. pernix group due to their morphological similarity and/or shallow genetic distances and highlight the importance of using as many types of data as possible in species descriptions.

However, I think the manuscript has a number of problems that should be addressed before it can be considered for publication. The main issues are listed below, section by section. There are also several minor issues (also listed below) involving wording, grammar, sentence structure, etc.

General:

Throughout the text, the authors consistently refer to the B. didactylus group, at one point even suggesting to provisionally transfer B. clarissae to that group. There is by now ample evidence (to the point that it is essentially a consensus) that this is an artificial, non-monophyletic group. Condez et al. (2020) and Folly et al. (2022) have suggested that the B. didactylus group should be no longer recognized, and this has been followed in subsequent studies (Mângia et al. 2023; Folly et al. 2024; Toledo et al. 2024), with the former members of that group being collectively referred to by the informal term “flea-toads”. I, too, recommend that the B. didactylus group be abandoned. Folly et al. (2022) also proposed to divide the former B. ephippium group in two monophyletic units: the B. ephippium and B. vertebralis groups (based on the presence/absence of a dorsal bony shield), which has also generally been followed.

Introduction

Line 46 - The Introduction starts a bit abruptly, addressing the species richness of the genus Brachycephalus out of nowhere. I think it would be appropriate to start the Introduction with a brief presentation of the genus, informing what group of amphibians it belongs to, its mode of reproduction, that it is endemic to the Brazilian Atlantic Forest, etc, before going into the details of how the genus grew in number of species through time.

Lines 83-92 – This paragraph should be transferred to the Materials and methods section, under a new subsection termed “Study area”. Also, more information on the Serra do Quiriri could be included here, such as geographic coordinates and data on mean annual temperature and rainfall.

Materials and methods

Lines 129-130 – I believe that in figure S1 the images of B and C are swapped, as the image in B shows warts that are much more densely packed than in C.

Line 231 – Why only those five species were used for the molecular analysis? And were the sequences for these species obtained from GenBank or generated by the authors for the present study?

Also, no mention is made of outgroups used in the analysis to root the tree.

Results

I think that the sections “Habitat and ecology”, “Geographical and altitudinal distribution” and “Remarks” could be united into a single section that could be called “Geographical distribution, habitat and natural history”. I see no point in keeping multiple sections with very little text in each of them.

Lines 473-474 – If the hand and foot are in palmar/plantar view, then it seems these are not the right hand and foot, but the left.

Lines 644-647 – The authors mention that the cestode was identified “due to the morphological similarity with parasites studied previously”. This is too vague. Was the parasite examined by a specialist? Was it deposited in a parasitological collection?

Also, it is briefly mentioned that there are few records of Ophiotaenia in amphibian hosts. No mention is made that one of these few records was from a closely related species (B. pernix) in which the cestodes were found inside the abdominal cavity (instead of subcutaneously as in B. lulai). I believe that this is an interesting fact and should be highlighted here.

Discussion

Lines 804-806 – Where did the authors get this hypothesis from? It goes somewhat counter to common knowledge. The theory of refugia and its derivations hypothesize that during glacial climatic cycles the forests tended to contract and be restricted to higher altitudes in the cold/dry phases and to expand towards the lowlands during the warm/wet phases (not the contrary). However, this scenario is usually applied to lower latitudes in S. America, such as the Amazonian region. I don’t know how this forest expansion/contraction dynamic has been estimated for more southern latitudes, in cooler climates. In any case, I think the authors should explain this hypothesis more clearly, citing their sources.

Lines 807-811 – The authors here hypothesize that B. lulai, B. aureoguttatus and B. quiririensis diverged from each other and speciated within the Serra do Quiriri through isolation in microrefugia. This assumption is based on the results of their phylogenetic analysis, that recovers these three species as a clade. However, as I remarked above in my comment on line 231, only six species (including B. lulai) have been included in the analysis, which may not provide a proper testing of the monophyly of what the authors call the “B. lulai clade” here. Indeed, most of the previous molecular phylogenies of the pernix group (e.g. Firkowski et al. 2016; Condez et al. 2021; Folly et al. 2022, 2024; Mângia et al. 2023), all of them using a much wider taxon coverage, have not recovered B. aureoguttatus and B. quiririensis as a clade. Thus, a more robust testing of the relationships of these two species (and, by extension, of B. lulai) with the other members of the pernix group should include a greater number of species in the ingroup, before any inferences regarding their speciation process can be made.

Lines 824-840 – I suggest that this whole paragraph be excluded and maybe the description of these forest types be transferred (in a shortened version) to the Materials and Methods section.

Minor issues:

Line 47 – Change “a small handful species diversity” to “only a handful of species”.

Line 50 – Change “from the aposematic coloration as in” to “from brightly colored, such as”.

Line 51 – Change “to the cryptic” to “to cryptically colored, such as”.

Lines 61-63 – Change “larger body sizes in comparison with members of the B. pernix group (up to 18.9 mm in snout-vent length against up to 15.8 mm in both groups, respectively)” to “larger body sizes (up to 18.9 mm in snout-vent length) in comparison with members of the B. pernix group (up to 15.8 mm)”.

Line 64 – Substitute “;” by “)”. Also, change “lacks” to “lack”.

Line 67 – Change “presence” to “absence”.

Lines 68-69 – The authors state that “current evidence suggests that B. didactylus species group is polyphyletic”. I consider that, by now, the evidence that this group is polyphyletic is quite solid, so that it would be more appropriate to say that it “strongly indicates” rather than just “suggests” that it is polyphyletic.

Lines 70-71 – Rewrite “Additional locations were surveyed and new species of Brachycephalus were uncovered. As a consequence, the known distribution of the genus expanded” as “As more localities were surveyed and new species were uncovered, the known distribution of the genus expanded…”

Line 83 – Change “edge” to “border”.

Line 88 – Change “also includes de occurrence of” to “is also inhabited by”

Line 89 – Change “the second from the type locality” to “the second only from the type locality”.

Line 90 – Change “distinguished” to “separated”.

Line 101 – Exclude “directly from their habitat”.

Lines 101-102 – Rewrite the text as “The advertisement calls aided the researchers in locating the males, whereas the females were collected haphazardly.”

Line 106 – Exclude “and each one identified”.

Line 108 – Change “euthanized” to “killed painlessly”.

Line 111 – Exclude “As part of the analysis,”.

Line 112 – Add “of various congeneric species” after “other specimens”.

Line 122 – Change “and taken by” to “and were taken by”.

Lines 128-129 – Change “warts at a density below a condition of side-by-side warts” to “warts that are not too close to each other”.

Line 130 – Change “warts side by side” to “warts that are densely packed, with little or no space between them”.

Line 133 – Add “of the holotype” after “the drawing”.

Line 154 – Change “Two transverse skin cuts in the medial and caudal regions” to “Two transverse skin cuts were taken from the medial and caudal regions”.

Line 160 – Change “performed” to “taken”.

Line 169 – Change “as Bornschein et al.” to “as in Bornschein et al.”.

Line 176 – Change “(notes per minute) (excluding attenuated notes)” to “(notes per minute, excluding attenuated notes)”.

Lines 178-179 – Change “notes of note groups” to “notes within note groups”.

Lines 188-189 – Change “(notes per minute) (calculated only when at least two note groups were present)” to “(notes per minute, calculated only when at least two note groups were present)”.

Line 250 – Exclude “and geographic distribution”.

Line 261 – Change “do not present a threat” to “do not seem to be threatened”.

Lines 288-289 – It is not necessary to describe here how the drawing was produced. This information was already given previously, in the MM section.

Line 290 – Add the word “by” after “Drawing”.

Lines 294 and 302 – Add the word “by” after “Photographs”.

Lines 310-311 – Since the paratypes 11593 and 10847 have the same date of collection, collectors and locality of the holotype, there is no need to repeat these data for each specimen. Instead, the information could be given as: “MHNCI 11593, male; MHNCI 10847, female, both collected with the holotype.

Lines 312-317 – There is also no need to repeat the data for males and females collected in the same day. Just mention all the specimens first (males and females, in this order) and then give the collection data, in each case. Ditto for the Paratypes on the next paragraph.

Line 332 – Change “is a member of…” to “is identified as a member of…”.

Line 336 – Delete the word “size”.

Line 343 – “small gray to dark irregular dots”. Dark is not a color!

Lines 351-353 – The new species can also be easily differentiated from these six species by color pattern and by the larger size of the females. This should be mentioned to improve the diagnosis.

Lines 365-366 – The authors mention a “bufoniform body shape” as one of the features not present in B. lulai”. However, in lines 333 and 336, B. lulai is said to have a bufoniform body shape.

Table 2 – On the “Color” column, substitute the word “points” by “dots” in all cases. Also, correct “diagnostics” to “diagnostic” in the legend

Lines 376-377 – I think this text should be in the legend of the table, not here.

Line 385 – For B. fuscolineatus, the locality data is given simply as “type locality”. It would be more informative to give the full locality data (as given for the other species) and then add the information that it is the type locality (e.g. by adding “; type locality” at the end).

Line 407 – Change “body sides” to “sides of body”.

Line 411 – I would substitute “reddish orange” with “uniform bright orange”.

Line 424 – Change “the females…” to “with females…”.

Line 431 – Change “becoming dark, brownish black” to “becoming dark brown or black”.

Line 442 – Change “maxillae” to “maxilla”. (maxillae is the plural form)

Line 444 – Change “The squamosal is” to “The squamosals are”.

Line 445 – Change “…towards to the maxillae; also have a long…” to “…towards the maxillae; they also have a long…”.

Line 455 – Change “fursed” to “fused”.

Lines 481 and 483 – Change “the medial and caudal of…” to “the medial and caudal portion of…”.

Line 536 – Rewrite “possibly to provide due the same reason that these calls…” as “possible to determine, since these calls…”.

Lines 555-556 – Change “in the number of 7–21 notes per call” to “with 7–21 notes per call”.

Line 582 – Change “The species is abundant” to “The species seems to be locally abundant”.

Line 584 – What does “later in the day” means? Late afternoon? Evening? Be more specific. If possible, give the hours at which calling activity was most intense (in case such data were recorded).

Line 586 – Change “Type locality of” to “Habitat at the type locality of”.

Line 588 – Change “East face of Pico Garuva, with 1,260 m” to “Eastern slope of Pico Garuva, at 1,260 m”.

Lines 597-598 – What do the green portions of the maps mean? Also, add the word “in” before “southeastern”.

Lines 625-629 – I suggest that the Table 7 should come after this paragraph, not before. Also, change “This conservation unit has a Weight of…” to “This conservation unit would have a Weight of…”, since the conservation unit does not exist yet.

Line 640 – Change “honor” to “honors” and “for three times president” to “who was for three times president”.

Line 641 – Change “and intent to stimulate” to “and intends to stimulate” or to “with the intent of stimulating”.

Line 644 – Change “and the chin in” to “and the gular region of”.

Line 645 – Correct “Ophiotenia” to “Ophiotaenia”. Also, did the authors count with the help of a specialist on helminths to help in the identification of the parasite? If so, please mention it and give the name of the specialist. If not, I suggest that the authors consult a specialist to confirm the identification (and, if possible, deposit it in a parasitological collection, giving the voucher number in the text).

Lines 649-650 – Change “parasitized by tapeworms of Ophiotaenia sp.” to “parasitized by tapeworms (Ophiotaenia sp.)”. Also, substitute “chin” by “gular region”.

Line 651 – Actually, the arrow in Fig. 13B is pointing to the frog’s right thigh, not the left.

Line 652 – Change “after dissection on the chin” to “after removal from the gular region”.

Line 653 – Add “individual” after “from the B. lulai.”.

Line 664 – The authors comment here on the degree of intraspecific genetic variability within species. However, no data on the intraspecific genetic variability is presented in the Results (or anywhere in the manuscript).

Line 684 – Change “Apart to” to “In addition to”.

Line 705 – Change “to issue” to “to emit”.

Line 725 – Change “to the own” to “to their own”.

Line 762 – Change “species of the B. pernix” to “species of the B. pernix group”.

Line 764 – Change “As the coloration in life” to “As for the coloration in life”.

Line 765 – Change “where not made” to “were not made”

Line 795 – Change “species boundary” to “species boundaries”.

Line 797 – Change “for increasingly” to “for increasing”.

Line 813 – Change “regions north of this domain” to “regions in the northern portion of this domain”.

Line 815 – What is “climax climatic over time”?

Lines 824-826 – The authors say here that “The arboreal vegetation that advances over the grasslands…is only ,<1 m high”. Can vegetation under 1 m high be considered arboreal?

Line 832 – Change “of the grow” to “of the growth”.

Line 851 – Change “phytotelma-breeding” to “phytotelm-breeding”.
