# Supplementary material for: A new species of Brachycephalus (Anura: Brachycephalidae) from Serra do Quiriri, northeastern Santa Catarina state, southern Brazil, with a review of the diagnosis among species of the B. pernix group and proposed conservation measures
Source: PLoS One. 2025 Dec 10;20(12):e0334746. doi: 10.1371/journal.pone.0334746 (PMC12694819; doi:10.1371/journal.pone.0334746)
Supplement: Attachment — Submitted filename: Rebuttal_letter.docx [file pone.0334746.s031.docx]

Curitiba, July 15, 2025

Dr. Neelesh Dahanukar,

Academic Editor

PLOS ONE

Dear Dr. Dahanukar,

We are grateful for the revisions we received and have carefully reviewed it to improve our manuscript. Addressing all comments required substantial word, which explains the delay in our resubmission. We also thoroughly the journal requirements and the Editor’s specific remarks. All revisions were made in a traceable manner for your review.

Below is our response to the reviewers, in bold for easy viewing. We numbered comments on our own, sequentially, so that we can direct reviewers to our responses in case of recurring comments.

Also, we inform, according journal requirements, that “*The funders had no role in study design, data collection and analysis, decision to publish, or preparation of the manuscript*”.

Kind regards,

Marcio Pie

______________________________________________________

**Reply to Journal Requirements**

1) Please ensure that your manuscript meets PLOS ONE's style requirements, including those for file naming. The PLOS ONE style templates can be found at:
*https://journals.plos.org/plosone/s/file?id=wjVg/PLOSOne_formatting_sample_main_body.pdf*
*https://journals.plos.org/plosone/s/file?id=ba62/PLOSOne_formatting_sample_title_authors_affiliations.pdf*

**Authors: Done.**

2) Thank you for stating the following financial disclosure:

“*The field work was funded by Fundação Grupo Boticário de Proteção à Natureza (through grant 1149_20191) through project conducted by Mater Natura – Instituto de Estudos Ambientais. MRP received a grant from CNPq/MCT (301636/2016-8). GS-S received grant from São Paulo Research Foundation (FAPESP; processes #2022/04847-7 and # 2023/09718-3). There was no additional external funding received for this study.*”

Please state what role the funders took in the study. If the funders had no role, please state: “*The funders had no role in study design, data collection and analysis, decision to publish, or preparation of the manuscript.*”

If this statement is not correct you must amend it as needed.

Please include this amended Role of Funder statement in your cover letter; we will change the online submission form on your behalf.

**Authors: Done (see above in our response).**

3) We note that you have indicated that there are restrictions to data sharing for this study. PLOS only allows data to be available upon request if there are legal or ethical restrictions on sharing data publicly. For more information on unacceptable data access restrictions, please see *http://journals.plos.org/plosone/s/data-availability#loc-unacceptable-data-access-restrictions*.

Before we proceed with your manuscript, please address the following prompts:

a) If there are ethical or legal restrictions on sharing a de-identified data set, please explain them in detail (e.g., data contain potentially identifying or sensitive patient information, data are owned by a third-party organization, etc.) and who has imposed them (e.g., a Research Ethics Committee or Institutional Review Board, etc.). Please also provide contact information for a data access committee, ethics committee, or other institutional body to which data requests may be sent.

b) If there are no restrictions, please upload the minimal anonymized data set necessary to replicate your study findings to a stable, public repository and provide us with the relevant URLs, DOIs, or accession numbers. For a list of recommended repositories, please see *https://journals.plos.org/plosone/s/recommended-repositories*. You also have the option of uploading the data as Supporting Information files, but we would recommend depositing data directly to a data repository if possible.

We will update your Data Availability statement on your behalf to reflect the information you provide.

**Authors: There may have been an error on our part if we indicated that there are restrictions on the data. The examined specimens and vocalizations are housed in public collections, and the list of materials, along with their catalog numbers, is provided in the manuscript as appendices. Osteological data are also deposited in an open-access repository, with the access link provided in the manuscript. Genetic data are available in GenBank, and the accession numbers have been included in the manuscript. The sequences were included as supplementary material.**

4) We note that Figure 11 and 12 in your submission contain map/satellite images which may be copyrighted. All PLOS content is published under the Creative Commons Attribution License (CC BY 4.0), which means that the manuscript, images, and Supporting Information files will be freely available online, and any third party is permitted to access, download, copy, distribute, and use these materials in any way, even commercially, with proper attribution. For these reasons, we cannot publish previously copyrighted maps or satellite images created using proprietary data, such as Google software (Google Maps, Street View, and Earth). For more information, see our copyright guidelines: *http://journals.plos.org/plosone/s/licenses-and-copyright*.

We require you to either (a) present written permission from the copyright holder to publish these figures specifically under the CC BY 4.0 license, or (b) remove the figures from your submission:

a. You may seek permission from the original copyright holder of Figure 11 and 12 to publish the content specifically under the CC BY 4.0 license.

We recommend that you contact the original copyright holder with the Content Permission Form (*http://journals.plos.org/plosone/s/file?id=7c09/content-permission-form.pdf*) and the following text:

“I request permission for the open-access journal PLOS ONE to publish XXX under the Creative Commons Attribution License (CCAL) CC BY 4.0 (*http://creativecommons.org/licenses/by/4.0/*). Please be aware that this license allows unrestricted use and distribution, even commercially, by third parties. Please reply and provide explicit written permission to publish XXX under a CC BY license and complete the attached form.”

Please upload the completed Content Permission Form or other proof of granted permissions as an “Other” file with your submission.

In the figure caption of the copyrighted figure, please include the following text: “Reprinted from [ref] under a CC BY license, with permission from [name of publisher], original copyright [original copyright year].”

b. If you are unable to obtain permission from the original copyright holder to publish these figures under the CC BY 4.0 license or if the copyright holder’s requirements are incompatible with the CC BY 4.0 license, please either i) remove the figure or ii) supply a replacement figure that complies with the CC BY 4.0 license. Please check copyright information on all replacement figures and update the figure caption with source information. If applicable, please specify in the figure caption text when a figure is similar but not identical to the original image and is therefore for illustrative purposes only.

The following resources for replacing copyrighted map figures may be helpful:

- USGS National Map Viewer (public domain): *http://viewer.nationalmap.gov/viewer/*
- The Gateway to Astronaut Photography of Earth (public domain): *http://eol.jsc.nasa.gov/sseop/clickmap/*
- Maps at the CIA (public domain): *https://www.cia.gov/library/publications/the-world-factbook/index.html* and *https://www.cia.gov/library/publications/cia-maps-publications/index.html*
- NASA Earth Observatory (public domain): *http://earthobservatory.nasa.gov/*
- Landsat: *http://landsat.visibleearth.nasa.gov/*
- USGS EROS (Earth Resources Observatory and Science (EROS) Center) (public domain): *http://eros.usgs.gov/#*
- Natural Earth (public domain): *http://www.naturalearthdata.com/*

**Authors: Done. The updated map now uses an OpenStreetMap basemap (ODbL-licensed), following the author guidelines**

5) We are unable to open your Supporting Information file S1_File.nex and doc.kml. Please kindly revise as necessary and re-upload.

**Authors: Done.**

6) Please take this opportunity to be sure you have met all of our guidelines for new species. For proper registration of a new zoological taxon, we require two specific statements to be included in your manuscript.

6.1. In the Results section, the globally unique identifier (GUID), currently in the form of a Life Science Identifier (LSID), should be listed under the new species name, for example:

*Anochetus boltoni* Fisher sp. nov. urn:*lsid:zoobank.org:act:B6C072CF-1CA6-40C7-8396-534E91EF7FBB*. Another LSID for the manuscript itself should also appear within the Nomenclature statement. You will need to contact Zoobank (*zoobank.org/About*) to obtain a GUID (LSID). You should receive one LSID for your manuscript and a separate, unique LSID for the new species.

6.2. Please also insert the following text into the Methods section, in a sub-section to be called “Nomenclatural Acts”:

The electronic edition of this article conforms to the requirements of the amended International Code of Zoological Nomenclature, and hence the new names contained herein are available under that Code from the electronic edition of this article. This published work and the nomenclatural acts it contains have been registered in ZooBank, the online registration system for the ICZN. The ZooBank LSIDs (Life Science Identifiers) can be resolved and the associated information viewed through any standard web browser by appending the LSID to the prefix “*http://zoobank.org/*”. The LSID for this publication is: urn:*lsid:zoobank.org:pub*: XXXXXXX. The electronic edition of this work was published in a journal with an ISSN, and has been archived and is available from the following digital repositories: PubMed Central, LOCKSS [author to insert any additional repositories].

All PLOS ONE articles are deposited in PubMed Central and LOCKSS. If your institute, or those of your co-authors, has its own repository, we recommend that you also deposit the published online article there and include the name in your article.

Following a recent ruling by the International Commission on Zoological Nomenclature, electronic journals are now a valid format for publication of new zoological taxa. In order to ensure the valid publication of your new species, please be sure to include the updated version of Nomenclatural Acts (above). A complete explanation of our guidelines for publishing new species can be found on our website: *http://www.plosone.org/static/guidelines#zoological*.

**Authors: Done.**

**______________________________________________________**

**Reply to Editor’s comments**

7.1) Defining the *Brachycephalus pernix* group: Authors mention the *B. pernix* group in the introduction without properly defining it and providing information about the number of species under this group. The references used for defining the group are old and several species have been published ever since. So there is a need to provide the number of species currently present in the group and also list them for easy reference to the readers.

**Authors: We defined the characteristics of the three groups in the second paragraph on the Introduction. Indeed, we had not mentioned the number of species in each group in the Introduction, but we did so in the Diagnosis section, with the appropriate references. We have now included the number of species in each group in the Introduction. However, we believe that listing the species belonging to each group would require bringing into the Introduction all the specific comments from the Diagnosis section regarding which author assigned each species described after 2015 to which group. This would disrupt the flow of the Introduction with highly technical content. In any case, we remain open to making this adjustment if the Editor still considers it beneficial for the readers.**

7.2) Based on Table 2, it seems that there are 20 species currently know in this species group [*B. pernix* group]. If this is the case, why does the number of species differ substantially for different types of analysis and comparison. For example, the genetic analysis is based on only six species, while the conservation analysis is for 12 species. Authors need to keep some consistency in the arguments and explain why other species are not considered for the given analysis.

**Authors: Regarding the number of species included in the genetic analysis, please see our response under Question #8.3. As for the species used in the conservation analysis, we have included the 12 species of the *B. pernix* group that occur in the state of Santa Catarina, since we are proposing the creation of a conservation unit in this state. We have now analyzed all 20 species of the *B. pernix* group.**

8.1) Phylogenetic analysis: Phylogenetic analysis is not presented properly.

**Authors: We thank the editor for the thoughtful suggestions on our molecular work. We now provide a more appropriate account of our goals and analyses. However, before we start, we want to emphasize that the validity of our new species, according to the ICZN, does not require any genetic data. We include it in our study to provide as much information as we can at this point about the new species.**

8.2) Authors are not allowed to pick and choose some species for phylogenetic analysis.

**Authors: We thank this opportunity to clarify this point. As will become clear below, our choice of species to be included in our analysis was far from arbitrary. However, we agree that the rationale for that choice was not explicit in our manuscript, and we added text to inform the reader about it.**

8.3) Molecular data are available for several species of *B. pernix* group and authors should use all available species in the group for analysis, even if some of the genetic markers are missing. In fact, it is advisable that authors use all member so the genus for larger phylogeny to show the monophyly of the group and then the taxonomic position of the new species.

**Authors: We understand this concern. However, including all of the genetic information for the group would not solve this issue, as we explain below.**

**The first comprehensive phylogeny of the *B. pernix* species group was provided by our research group in 2016 (Firkowski et al. 2016) and included only four loci through Sanger sequencing (i.e. 16S, cytb, ND2, and RPL). This was followed up by a phylogenomic study using UCEs and thousands of loci (Pie et al. 2018). There is only one locus in common (16S) between the current study and Firkowski et al. (2016), and there is no UCE data for the new species. Therefore, there would not be any shared information to place the new species beyond 16S. In addition, given the vast difference in the sizes of the datasets, in practice that would be tantamount to imposing topological constraints of the tree obtained with the phylogenomic dataset, which had very high support. In other words, it would be impossible for a single locus to overcome the influence of hundreds of UCE loci, and we would end up with the same tree as the UCE tree, except that the new species would be placed only with 16S. We believe that a more reasonable approach would be to use the phylogenomic analysis to inform our choice of taxa and rooting, as now made more explicit in the text, as follows. In the methods section, we wrote:**

**“To determine the phylogenetic position of the new species, we analyzed DNA sequences from four paratypes (MHNCI 11596, MHNCI 11598–11600).** **The choice of species to be analyzed alongside the new species was based on previous work on the phylogeny of the group (i.e. Firkowski et al. 2016, Pie et al. 2018). Preliminary analyses indicated that the new species was closely related to a well-supported clade that included *B. auroguttatus*, *B. quiririensis*, *B. ferruginus*, *B. pernix*, and *B. pombali*. The monophyly of this clade was first suggested by Firkowski et al. (2016) using Sanger sequencing and was later strongly supported by a phylogenomic analysis using UCEs by Pie et al. (2018). We therefore chose those species for exploring the phylogenetic position of the new species. The resulting tree was rooted according to the phylogenomic work by Pie et al. (2018).”**

**In the results section, we rewrote the following:**

***Phylogenetic relationships***

**The inferred phylogenetic relationships and phylogenetic distances between *B. lulai* and other species of the *B. pernix* species group are shown Fig 9 and Table 6, respectively. All specimens of *B. lulai* form a clade with strong support, which itself forms a larger clade together with *B. auroguttatus* and *B. quiririensis*, also with strong support…**

8.4) The genetic analysis is also not presented properly.

**Authors: We now provide more information (see above).**

8.5) The outgroups are not defined.

**Authors: As indicated above, the tree was rooted according to the relationships found in the phylogenomic study by Pie et al. (2018).**

8.6) There are large branches within the clades and there are politomies, suggesting that the trees are not resolved.

**Authors: We are not sure what is meant by large branches. If phylogenies are generated with only a few Sanger loci, such a level of unevenness in distances from the root to the tips is very common (we could provide dozens of examples). Indeed, the relationships found for the remaining species are largely the same as those found in a previous phylogenomic study using hundreds of loci, which suggests that there is nothing inherently wrong with our analyses.**

**Also, it is very common for a phylogeny to have varying levels of support between nodes, and it is up to the researchers to correctly interpret that phylogenetic uncertainty. In our case, none of the polytomies affects our conclusions that (1) all samples of *B. lulai* are monophyletic with strong support; (2) *B. lulai* forms a clade with *B. auroguttatus* and *B. quiririensis* (even though the relationships among these three species cannot be ascertained at the moment). Of those results, monophyly of the samples of *B. lulai* seems to us the most important result, and that conclusion is robust given our analyses.**

8.7) Authors should increase the taxonomic sampling and perform maximum likelihood analysis in addition to Bayesian analysis.

**Authors: In the methods section, we added the following text:**

**“****Additional analyses using maximum likelihood provided qualitatively identical results and will not be shown for the sake of brevity”.**

**Just to illustrate, a ML tree is provided below with largely the same topology as the BI tree:**

8.8) The genetic distances provided by the authors make very little sense. If there are multiple individuals then there will be a range of genetic distance and not just one value. It is essential to provide this range (minimum-maximum) for the intra and inter species genetic distance. I will refrain from providing the mean as the number of individuals are low and even if means are provided they should be accompanied with the standard deviation or some measure of uncertainty.

**Authors: This is now included in the revised version of the text.**

8.9) The long branches in the proposed species are worrisome, and it is essential that authors prove that the new species is genetically distinct with species delimitation methods such as barcode gap (using ASAP) and Poisson tree process.

**Authors: Given that our text is already long for a species description, we would rather not delve into this subject, given that we believe that species delimitation methods using only a few loci are largely problematic. Our goal was not to use genetic data for species delimitation, given that we believe that the new species is well supported based on several independent phenotypic characters, and our goal was simply to provide an approximate phylogenetic placement of the new species and to indicate that all samples of the new species are monophyletic. Indeed, when we used only a few loci in Firkowski et al. (2016), some the *Brachycephalus* species were not monophyletic using Sanger loci and would therefore be considered as invalid based on ASAP or PTP, yet they were highly supported when using hundreds of UCE loci and a more powerful method of species delimitation (i.e. BPP, Pie et al. [2018]).**

9) Conservation status: This is the weakest part of the manuscript. Authors are using methodology that they themselves have devised for defining IUCN categories and they are not referring to proper IUCN guidelines for defining the categories based on the various criteria that are defined by the IUCN Redlist of Threatened Taxa documentation. If authors are using their own methodologies, there is no point in using the IUCN categories for defining the conservation status. The IUCN guidelines provides proper rationale for when to use different categories based on five different criteria used for assessments. Author's argument that the species is DD as a polygon cannot be constructed is flawed. The point localities are enough to define the Area of Occupancy based on 2km grids and have been used for defining the conservation status where the species are point endemics. Either authors should understand how the various criteria are used for finding the IUCN categories for red listing or authors should not used the categories used by the IUCN. In the current case if the threats to the species are know and the distribution is known, the species is not DD.

**Authors: Thank you. We followed IUCN criteria and removed the adaptations in the status assessment that were previously made due to the particularities of the species distribution.**

**______________________________________________________**

**Reply to Reviewer’s comments**

10) Throughout the text, the authors consistently refer to the *B. didactylus* group, at one point even suggesting to provisionally transfer *B. clarissae* to that group. There is by now ample evidence (to the point that it is essentially a consensus) that this is an artificial, non-monophyletic group. Condez et al. (2020) and Folly et al. (2022) have suggested that the *B. didactylus* group should be no longer recognized, and this has been followed in subsequent studies (Mângia et al. 2023; Folly et al. 2024; Toledo et al. 2024), with the former members of that group being collectively referred to by the informal term “flea-toads”. I, too, recommend that the *B. didactylus* group be abandoned. Folly et al. (2022) also proposed to divide the former B. ephippium group in two monophyletic units: the *B. ephippium* and *B. vertebralis* groups (based on the presence/absence of a dorsal bony shield), which has also generally been followed.

**Authors: We understand and follow all these discussions, but it is important to note, as the reviewer rightly pointed out, that the cited authors are referring to a very well-defined group of species based on morphological characteristics—so solid and well-established that it earned the popular name “flea-toads”. The cited authors are using a new terminology to replace one of the phenetic groups previously proposed: *B. didactylus*. We agree that the *B. didactylus* group, or the “flea-toads” group, is polyphyletic, and we make that very clear in the text—especially in this revised version (see question #28). However, with all due respect, we do not find it appropriate to replace a categorically proposed term with an equivalent that is used loosely and without formal definition to refer to a set of similar species in formal species descriptions.**

**As for *B. clarissae*, we have rewritten the association of this species with the *B. didactylus* group, citing, now, Toledo et al. (2024). Although these authors did not use the term *B. didactylus* group, they included *B. clarissae* among all *Brachycephalus* species with a “leptodactyliform” body shape, which is the diagnostic characteristic. Therefore, since there is no error in adopting the phenetic group proposal by Ribeiro et al. (2015), and considering that the *B. didactylus* group continues to be used as a phenetic group—even if renamed as “flea-toads”—we prefer to maintain the use of the *B. didactylus* group as proposed by Ribeiro et al. (2015).**

11) Introduction - Line 46 - The Introduction starts a bit abruptly, addressing the species richness of the genus *Brachycephalus* out of nowhere. I think it would be appropriate to start the Introduction with a brief presentation of the genus, informing what group of amphibians it belongs to, its mode of reproduction, that it is endemic to the Brazilian Atlantic Forest, etc, before going into the details of how the genus grew in number of species through time.

**Authors: Done.**

12) Introduction - Lines 83-92 – This paragraph should be transferred to the Materials and methods section, under a new subsection termed “Study area”. Also, more information on the Serra do Quiriri could be included here, such as geographic coordinates and data on mean annual temperature and rainfall.

**Authors: Done.**

13) Materials and methods - Lines 129-130 – I believe that in figure S1 the images of B and C are swapped, as the image in B shows warts that are much more densely packed than in C.

**Authors: Thank you for the observation. We have corrected the figure.**

14) Materials and methods - Line 231 – Why only those five species were used for the molecular analysis? And were the sequences for these species obtained from GenBank or generated by the authors for the present study?

Also, no mention is made of outgroups used in the analysis to root the tree.

**Authors: Please, see our detailed responses about this matter under question #8.**

15) Results - I think that the sections “Habitat and ecology”, “Geographical and altitudinal distribution” and “Remarks” could be united into a single section that could be called “Geographical distribution, habitat and natural history”. I see no point in keeping multiple sections with very little text in each of them.

**Authors: Done.**

16) Results - Lines 473-474 – If the hand and foot are in palmar/plantar view, then it seems these are not the right hand and foot, but the left.

**Authors:** **Thank you for the observation. In fact, Figure 6 shows in plantar view the left hand in G and the left foot in H. We proceeded with the correction of the caption.**

17) Results - Lines 644-647 – The authors mention that the cestode was identified “due to the morphological similarity with parasites studied previously”. This is too vague. Was the parasite examined by a specialist? Was it deposited in a parasitological collection?

**Authors:** **The parasites were found under the same conditions, without obvious morphological differences and in a immature stage of development. Therefore, there is no way to identify the parasite through morphological traits. We consulted with Dr. Karla Magalhães Campião, from the Departamento de Zoologia at the Universidade Federal do Paraná, a amphibian parasite specialist, who confirmed the lack of morphological structures for identification. In a previous study cited in the manuscript, the parasite genus was identified through a phylogenetic tree obtained by maximum parsimony analysis using the nuclear 18S and 28S rDNA gene, in which three authors of the present manuscript are also authors of this previous study. To date, we have not deposited the parasites in a parasitological collection, as we intend to continue studying this interaction. However, to underscore the current uncertainty in this identification by stating that “We tentatively identified these as *Ophiotaenia* sp.”**

18) Results - Also, it is briefly mentioned that there are few records of Ophiotaenia in amphibian hosts. No mention is made that one of these few records was from a closely related species (B. pernix) in which the cestodes were found inside the abdominal cavity (instead of subcutaneously as in B. lulai). I believe that this is an interesting fact and should be highlighted here.

**Authors:** **We address this question in a new text in the Remarks section.**

19) Discussion - Lines 804-806 – Where did the authors get this hypothesis from? It goes somewhat counter to common knowledge. The theory of refugia and its derivations hypothesize that during glacial climatic cycles the forests tended to contract and be restricted to higher altitudes in the cold/dry phases and to expand towards the lowlands during the warm/wet phases (not the contrary). However, this scenario is usually applied to lower latitudes in S. America, such as the Amazonian region. I don’t know how this forest expansion/contraction dynamic has been estimated for more southern latitudes, in cooler climates. In any case, I think the authors should explain this hypothesis more clearly, citing their sources.

**Authors: With all due respect, there are many studies reporting that, in southern Brazil, in cold and dry conditions, forests occurred at lower altitudes and, with greater rainfall and increased heat, reached higher altitudes, overtaking the grasslands (e.g., Behling’s works). In the proposed biogeographic hypothesis, which was duly cited (articles #15, 33, and 65), these studies were explored. If the reviewer deems it necessary, we will incorporate this literature into the discussion, but we emphasize that we were brief on this aspect of the theory in order to advance an unprecedented discussion on structural aspects of vegetation in this dynamism.**

20) Discussion - Lines 807-811 – The authors here hypothesize that *B. lulai*, *B. aureoguttatus* and *B. quiririensis* diverged from each other and speciated within the Serra do Quiriri through isolation in microrefugia. This assumption is based on the results of their phylogenetic analysis, that recovers these three species as a clade. However, as I remarked above in my comment on line 231, only six species (including *B. lulai*) have been included in the analysis, which may not provide a proper testing of the monophyly of what the authors call the “*B. lulai* clade” here. Indeed, most of the previous molecular phylogenies of the *pernix* group (e.g. Firkowski et al. 2016; Condez et al. 2021; Folly et al. 2022, 2024; Mângia et al. 2023), all of them using a much wider taxon coverage, have not recovered *B. aureoguttatus* and *B. quiririensis* as a clade. Thus, a more robust testing of the relationships of these two species (and, by extension, of *B. lulai*) with the other members of the *pernix* group should include a greater number of species in the ingroup, before any inferences regarding their speciation process can be made.

**Authors: We provided detailed the support for the monophyly of *B. lulai*, as well as for the clade formed by this species with *B. auroguttatus* and *B. quiririensis* under the question #8. There, we also highlighted that the relationships among these three species remain unresolved; however, this does not prevent the hypothesis of speciation from being proposed.**

21) Discussion - Lines 824-840 – I suggest that this whole paragraph be excluded and maybe the description of these forest types be transferred (in a shortened version) to the Materials and Methods section.

**Authors: This paragraph contains the most important discussion in the manuscript. However, we agree that the previous wording was not sufficiently clear and gave the impression of being a detached description of vegetation, without highlighting the crucial point to be discussed. We have therefore rewritten a substantial portion of the biogeographic discussion, aiming to make the argument significantly more assertive.**

22) Line 47 – Change “a small handful species diversity” to “only a handful of species”.

**Authors: Done. We adjusted the sentence, but replaced “a handful of” with “a few” to avoid potential confusion suggesting there were only five species.**

23) Line 50 – Change “from the aposematic coloration as in” to “from brightly colored, such as”.

**Authors: Done.**

24) Line 51 – Change “to the cryptic” to “to cryptically colored, such as”.

**Authors: Done.**

25) Lines 61-63 – Change “larger body sizes in comparison with members of the *B. pernix* group (up to 18.9 mm in snout-vent length against up to 15.8 mm in both groups, respectively)” to “larger body sizes (up to 18.9 mm in snout-vent length) in comparison with members of the *B. pernix* group (up to 15.8 mm)”.

**Authors: Done.**

26) Line 64 – Substitute “;” by “)”. Also, change “lacks” to “lack”.

**Authors: Done.**

27) Line 67 – Change “presence” to “absence”.

**Authors: Done.**

28) Lines 68-69 – The authors state that “current evidence suggests that *B. didactylus* species group is polyphyletic”. I consider that, by now, the evidence that this group is polyphyletic is quite solid, so that it would be more appropriate to say that it “strongly indicates” rather than just “suggests” that it is polyphyletic.

**Authors: Done.**

29) Lines 70-71 – Rewrite “Additional locations were surveyed and new species of *Brachycephalus* were uncovered. As a consequence, the known distribution of the genus expanded” as “As more localities were surveyed and new species were uncovered, the known distribution of the genus expanded…”

**Authors: Done.**

30) Line 83 – Change “edge” to “border”.

**Authors: Done.**

31) Line 88 – Change “also includes de occurrence of” to “is also inhabited by”

**Authors: Done.**

32) Line 89 – Change “the second from the type locality” to “the second only from the type locality”.

**Authors: Done.**

33) Line 90 – Change “distinguished” to “separated”.

**Authors: Done.**

34) Line 101 – Exclude “directly from their habitat”.

**Authors: Done.**

35) Lines 101-102 – Rewrite the text as “The advertisement calls aided the researchers in locating the males, whereas the females were collected haphazardly.”

**Authors: Done.**

36) Line 106 – Exclude “and each one identified”.

**Authors: Done.**

37) Line 108 – Change “euthanized” to “killed painlessly”.

**Authors: Done.**

38) Line 111 – Exclude “As part of the analysis,”.

**Authors: Done.**

39) Line 112 – Add “of various congeneric species” after “other specimens”.

**Authors: Done.**

40) Line 122 – Change “and taken by” to “and were taken by”.

**Authors: Done.**

41) Lines 128-129 – Change “warts at a density below a condition of side-by-side warts” to “warts that are not too close to each other”.

**Authors: Done.**

42) Line 130 – Change “warts side by side” to “warts that are densely packed, with little or no space between them”.

**Authors: Done.**

43) Line 133 – Add “of the holotype” after “the drawing”.

**Authors: Done.**

44) Line 154 – Change “Two transverse skin cuts in the medial and caudal regions” to “Two transverse skin cuts were taken from the medial and caudal regions”.

**Authors: Done.**

45) Line 160 – Change “performed” to “taken”.

**Authors: Done.**

46) Line 169 – Change “as Bornschein et al.” to “as in Bornschein et al.”.

**Authors: Done.**

47) Line 176 – Change “(notes per minute) (excluding attenuated notes)” to “(notes per minute, excluding attenuated notes)”.

**Authors: Done.**

48) Lines 178-179 – Change “notes of note groups” to “notes within note groups”.

**Authors: Done.**

49) Lines 188-189 – Change “(notes per minute) (calculated only when at least two note groups were present)” to “(notes per minute, calculated only when at least two note groups were present)”.

**Authors: Done.**

50) Line 250 – Exclude “and geographic distribution”.

**Authors: Done.**

51) Line 261 – Change “do not present a threat” to “do not seem to be threatened”.

**Authors: Done.**

52) Lines 288-289 – It is not necessary to describe here how the drawing was produced. This information was already given previously, in the MM section.

**Authors: Done.**

53) Line 290 – Add the word “by” after “Drawing”.

**Authors: Done.**

54) Lines 294 and 302 – Add the word “by” after “Photographs”.

**Authors: Done.**

55) Lines 310-311 – Since the paratypes 11593 and 10847 have the same date of collection, collectors and locality of the holotype, there is no need to repeat these data for each specimen. Instead, the information could be given as: “MHNCI 11593, male; MHNCI 10847, female, both collected with the holotype.

**Authors: Done.**

56) Lines 312-317 – There is also no need to repeat the data for males and females collected in the same day. Just mention all the specimens first (males and females, in this order) and then give the collection data, in each case. Ditto for the Paratypes on the next paragraph.

**Authors: Done.**

57) Line 332 – Change “is a member of…” to “is identified as a member of…”.

**Authors: Done.**

58) Line 336 – Delete the word “size”.

**Authors: Done.**

59) Line 343 – “small gray to dark irregular dots”. Dark is not a color!

**Authors: Done.**

60) Lines 351-353 – The new species can also be easily differentiated from these six species by color pattern and by the larger size of the females. This should be mentioned to improve the diagnosis.

**Authors:** With all due respect, there may have been a misunderstanding, either in the reviewer’s comment or on our part. We were unable to identify the passage in the text that was referenced. In any case, we would like to emphasize that detailed information regarding the diagnostic characteristics of B. lulai, which appears to be the reviewer’s concern, is indeed provided in the text**.**

61) Lines 365-366 – The authors mention a “bufoniform body shape” as one of the features not present in *B. lulai*”. However, in lines 333 and 336, *B. lulai* is said to have a bufoniform body shape.

**Authors: Done. We rewrote the text and corrected the information that was, in fact, incorrect.**

62) Table 2 – On the “Color” column, substitute the word “points” by “dots” in all cases. Also, correct “diagnostics” to “diagnostic” in the legend

**Authors: Done.**

63) Lines 376-377 – I think this text should be in the legend of the table, not here.

**Authors: Done.**

64) Line 385 – For *B. fuscolineatus*, the locality data is given simply as “type locality”. It would be more informative to give the full locality data (as given for the other species) and then add the information that it is the type locality (e.g. by adding “; type locality” at the end).

**Authors: Done.**

65) Line 407 – Change “body sides” to “sides of body”.

**Authors: Done.**

66) Line 411 – I would substitute “reddish orange” with “uniform bright orange”.

**Authors: Done.**

67) Line 424 – Change “the females…” to “with females…”.

**Authors: Done.**

68) Line 431 – Change “becoming dark, brownish black” to “becoming dark brown or black”.

**Authors: Done.**

69) Line 442 – Change “maxillae” to “maxilla”. (maxillae is the plural form)

**Authors: Done.**

70) Line 444 – Change “The squamosal is” to “The squamosals are”.

**Authors: Done.**

71) Line 445 – Change “…towards to the maxillae; also have a long…” to “…towards the maxillae; they also have a long…”.

**Authors: Done.**

72) Line 455 – Change “fursed” to “fused”.

**Authors: Done.**

73) Lines 481 and 483 – Change “the medial and caudal of…” to “the medial and caudal portion of…”.

**Authors: Done.**

74) Line 536 – Rewrite “possibly to provide due the same reason that these calls…” as “possible to determine, since these calls…”.

**Authors: Done.**

75) Lines 555-556 – Change “in the number of 7–21 notes per call” to “with 7–21 notes per call”.

**Authors: Done.**

76) Line 582 – Change “The species is abundant” to “The species seems to be locally abundant”.

**Authors: Done.**

77) Line 584 – What does “later in the day” means? Late afternoon? Evening? Be more specific. If possible, give the hours at which calling activity was most intense (in case such data were recorded).

**Authors: Done. We rewrote the sentence, including the time information.**

78) Line 586 – Change “Type locality of” to “Habitat at the type locality of”.

**Authors: Done.**

79) Line 588 – Change “East face of Pico Garuva, with 1,260 m” to “Eastern slope of Pico Garuva, at 1,260 m”.

**Authors: Done.**

80) Lines 597-598 – What do the green portions of the maps mean? Also, add the word “in” before “southeastern”.

**Authors: Done. We have included this information in the figure caption.**

81) Lines 625-629 – I suggest that the Table 7 should come after this paragraph, not before. Also, change “This conservation unit has a Weight of…” to “This conservation unit would have a Weight of…”, since the conservation unit does not exist yet.

**Authors: Done.**

82) Line 640 – Change “honor” to “honors” and “for three times president” to “who was for three times president”.

**Authors: Done. However, we added “elected” before “was”, since he is still the president.**

83) Line 641 – Change “and intent to stimulate” to “and intends to stimulate” or to “with the intent of stimulating”.

**Authors: Done.**

84) Line 644 – Change “and the chin in” to “and the gular region of”.

**Authors: Done.**

85) Line 645 – Correct “Ophiotenia” to “Ophiotaenia”. Also, did the authors count with the help of a specialist on helminths to help in the identification of the parasite? If so, please mention it and give the name of the specialist. If not, I suggest that the authors consult a specialist to confirm the identification (and, if possible, deposit it in a parasitological collection, giving the voucher number in the text).

**Authors: We corrected the text (please, see under question #17).**

86) Lines 649-650 – Change “parasitized by tapeworms of *Ophiotaenia* sp.” to “parasitized by tapeworms (*Ophiotaenia* sp.)”. Also, substitute “chin” by “gular region”.

**Authors: Done.**

87) Line 651 – Actually, the arrow in Fig. 13B is pointing to the frog’s right thigh, not the left.

**Authors: Done. We corrected the arrows in the figure.**

88) Line 652 – Change “after dissection on the chin” to “after removal from the gular region”.

**Authors: Done.**

89) Line 653 – Add “individual” after “from the *B. lulai*.”.

**Authors: Done.**

90) Line 664 – The authors comment here on the degree of intraspecific genetic variability within species. However, no data on the intraspecific genetic variability is presented in the Results (or anywhere in the manuscript).

**Authors: This information is now present in the new version of Table 6.**

91) Line 684 – Change “Apart to” to “In addition to”.

**Authors: Done.**

92) Line 705 – Change “to issue” to “to emit”.

**Authors: Done.**

93) Line 725 – Change “to the own” to “to their own”.

**Authors: Done.**

94) Line 762 – Change “species of the B. *pernix*” to “species of the B. *pernix* group”.

**Authors: Done.**

95) Line 764 – Change “As the coloration in life” to “As for the coloration in life”.

**Authors: Done.**

96) Line 765 – Change “where not made” to “were not made”

**Authors: Done.**

97) Line 795 – Change “species boundary” to “species boundaries”.

**Authors: Done.**

98) Line 797 – Change “for increasingly” to “for increasing”.

**Authors: Done.**

99) Line 813 – Change “regions north of this domain” to “regions in the northern portion of this domain”.

**Authors: Done.**

100) Line 815 – What is “climax climatic over time”?

**Authors: Done. We rewrote the sentence to make it clear.**

101) Lines 824-826 – The authors say here that “The arboreal vegetation that advances over the grasslands…is only ,<1 m high”. Can vegetation under 1 m high be considered arboreal?

**Authors: Done. Very well observed. We rewrote the sentence and corrected the error.**

102) Line 832 – Change “of the grow” to “of the growth”.

**Authors: Done.**

103) Line 851 – Change “phytotelma-breeding” to “phytotelm-breeding”.

**Authors: Done.**
