# Supplementary material for: A new species of Brachycephalus (Anura: Brachycephalidae) from Serra do Quiriri, northeastern Santa Catarina state, southern Brazil, with a review of the diagnosis among species of the B. pernix group and proposed conservation measures
Source: PLoS One. 2025 Dec 10;20(12):e0334746. doi: 10.1371/journal.pone.0334746 (PMC12694819; doi:10.1371/journal.pone.0334746)
Supplement: Attachment — Submitted filename: Response to reviewers_R2 -September.docx [file pone.0334746.s032.docx]

Curitiba, September 20, 2025

Dr. Neelesh Dahanukar,

Academic Editor

PLOS ONE

Dear Dr. Dahanukar,

**We thank the reviewer for their thorough analysis of the entire manuscript, down to its smallest details. We have accepted all suggestions, except for the one regarding a more detailed explanation of the etymology of the new species. This is because we have chosen to simplify the etymology, and therefore the suggested addition is no longer necessary.**

**Despite fully accepting all other points, we have still chosen to respond to each comment individually, in order to highlight the breadth of the review.**

**Below, you will find our responses to the reviewer’s comments, presented in bold for ease of reading. We have numbered the comments sequentially ourselves, so that we can refer back to our responses in the case of recurring issues.**

Kind regards,

Marcio Pie

**Reply to Reviewer’s comments**

1) As stated in my first review of this ms, I still discourage the continuing use of the *B. didactylus* group as a taxonomic entity. The authors have responded to this critic by arguing that the *B. didactylus* group is “a very well-defined group of species based on morphological characteristics”. Species groups are treated in current zoological systematics as monophyletic groupings of species rather than purely phenetic ones (unless when the phylogenetic relationships among species are not known). And it is now widely known that the characteristics uniting the species in the *B. didactylus* group (including *B. clarissae*, which does not share all of them) are plesiomorphic for the genus. Yet, even after addressing the non-monophyly of the *B. didactylus* group in the introduction, the authors continue to refer to it throughout the text as if it was a taxonomic entity equivalent to the other two species groups in the genus (for which there is strong evidence of monophyly in the literature). I consider this misleading, and continue to advocate for the use of the term “flea toads” instead, which unlike the “species group” category, corresponds to a morphotype rather than a clade. Indeed, the term is adopted in the work of Toledo et al. (2024), which *included B. clarissae* among the species with that morphotype (as mentioned by the authors in their response to my previous review).

The authors also seem to use the *B. ephippium* group in the sense of Ribeiro et al. 2015 (i.e. including both the ephippium and vertebralis lineages proposed by Condez et al. 2020) rather than in the sense of Folly et al. 2022 (i.e. excluding the vertebralis lineage, which is considered as a separate species group). The latter definition seems more conservative, since not all phylogenetic analyses published so far presented strong support for the monophyly of the B. ephippium group sensu Ribeiro et al. 2015. Anyway, since there are currently two different definitions of this group being used in the literature, the authors should explicitly state in the text which one they are using.

**Authors: DONE.**

**We have started using “flea toads” instead of the “*B. didactylus* group”. In the case of the *B. ephippium* group, we have taken the liberty of continuing to adopt the original proposal by Ribeiro et al. (2015). Since the new species described belongs to the *B. pernix* group, it seems more didactic and understandable to diagnose it in relation to the *B. ephippium* group *lato sensu*.**

**Perhaps the reviewer did not notice that we made explicit which proposal we adopted regarding the *B. ephippium* group in the following passage of the diagnosis: “*Brachycephalus lulai* sp. nov. can be distinguished from the seven flea toad species (…) by having bufoniform body shape instead leptodactyliform body shape (…) and from 15 species of the *B. ephippium* group (sensu Ribeiro et al. [13]; …”**

2) I believe that in all tables, as well as in table and figure legends that feature the name “*B. lulai*”, it should be followed by “sp. nov.”

**Authors: DONE.**

**However, we standardized by adding “sp. nov.” to every mention of B. lulai.**

3) Line 4 – I suggest adding the word “proposed” before “conservation measures”.

**Authors: DONE.**

4) Line 26 – Exclude the hyphen in “currently-recognized”.

**Authors: DONE.**

5) Line 29 – Change “call comparisons” to “acoustic comparisons”.

**Authors: DONE.**

6) Line 52 – “The species are diurnal”. This should be changed to “…mostly diurnal”, since some of the cryptically colored “flea toads” often present nocturnal activity.

**Authors: DONE.**

7) Line 98 – Change “also inhabited de occurrence of” to “also inhabited by”.

**Authors: DONE.**

8) Line 102 – Change “occurs *B. leopardus*, a species known from two localities” to “*B. leopardus* occurs, being known from two localities”.

**Authors: DONE.**

9) Lines 106-107 – Change “annual mean of temperature” to “mean annual temperature”.

**Authors: DONE.**

10) Line 306 – Change “we did not mapped” to “we have not mapped”.

**Authors: DONE.**

11) Line 468 – “discrete irregular greenish or brown spots”. Do you mean “discrete” or “discreet”? These are different terms.

**Authors: DONE**

**We did mean “discrete”.**

12) Line 479 – Add the word “species” after “Sexually dimorphic”.

**Authors: DONE.**

13) Line 483 – Change “green spots” to “green patches”.

**Authors: We referred to the spots of *B. lulai* as “spots” seven times, and never as “patches.” Replacing only one instance of “spots” with “patches” could introduce ambiguity in the interpretation of the color pattern. Moreover, replacing all seven occurrences of “spots” with “patches” did not seem appropriate to us. That said, we remain open to adjusting the terminology used to describe the markings, depending on the reviewer’ suggestions.**

14) Line 486 – Change “becoming dark, brownish black” to “becoming dark gray”.

**Authors: DONE.**

15) Line 549 – “in B and C, von Kossa staining”. I believe there is an error here: it should be “B and D”, right?

**Authors: DONE**

**Exactly. Thank you very much for noticing this error.**

16) Line 643 – Change “in the southeastern hillside from” to “on the southeastern hillside of”.

**Authors: DONE.**

17) Line 645 – Change “it is likely that the *B. lulai* populations” to “it is likely that *B. lulai* populations”.

**Authors: DONE.**

18) Line 652 – Exclude the word “with”.

**Authors: DONE.**

19) Lines 662-663 – Change “of *B. lulai*” to “of one individual of *B. lulai*”.

**Authors: DONE.**

20) Lines 664-665 – Change “previously studied parasites [58] and found under the same conditions” to “previously recorded parasites found under similar conditions [58]”.

**Authors: DONE.**

21) Line 665 – Change “without apparent morphological structures and in a young stage” to “without apparent diagnostic morphological structures and at a young stage”.

**Authors: DONE.**

22) Lines 667 – Add the word “presumably” before “makes”.

**Authors: DONE.**

23) Lines 668-670 – This sentence starting with “Furthermore…” is confusing and it is not clear what it is trying to say. I suggest deleting this sentence and replacing it with this: “Indeed, *Ophiotaenia* spp. are generalist parasites that use not only amphibians, but also snakes and turtles as hosts [58].”

**Authors: DONE.**

24) Line 671 – Change “the *Ophiotaenia* parasite was found in” to “*Ophiotaenia* parasites have been found in”.

**Authors: DONE.**

25) Lines 671-672 – I think the record of *Ophiotaenia* in *B. pernix* by Ribeiro et al. (2014) should be cited here too.

**Authors: DONE.**

**Perhaps the reviewer did not notice, but the work by Ribeiro et al. (2014) was indeed cited [...a proteocephalid tapeworm, due to the morphological similarity with previously recorded parasites found under similar conditions [58],...].**

26) Line 676 – Change “of the parasite at the gular region” to “of the parasites in the gular region”.

**Authors: DONE.**

27) Line 692 – Change “even with the small distribution.” To “even considering its small distribution area.”

**Authors: DONE.**

28) Lines 693 and 699-700 – Change “From the 20 remaining species” to “Of the 20 remaining species”.

**Authors: DONE.**

29) Line 706 – Add the word “states” after “Santa Catarina”.

**Authors: DONE.**

30) Table 7 – In the line of *B. pombali*, change 1300 to 1,300.

**Authors: DONE.**

31) Line 735 – After “digital reduction”, I suggest adding “(President Lula lost the little finger on his left hand in an accident)”. I think an explanation is appropriate, since this pun may not be obvious for some non-Brazilian readers.

**Authors: As mentioned above, we opted to simplify the etymology, so the reviewer’ suggestion is no longer applicable.**

32) Line 749 – Change “support our interpretation” to “reinforce our interpretation”.

**Authors: DONE.**

33) Line 759 – Change “vertebrae” to “vertebra”.

**Authors: DONE.**

34) Lines 783 and 784 - Change “other words” to “other works”.

**Authors: DONE.**

35) Line 784 – Change “described to as” to “described as”.

**Authors: DONE.**

36) Line 819 – Change “did not note this feature” to “did not mention this feature”.

**Authors: DONE.**

37) Line 825 – Change “latter it was demonstrated” to “it was later demonstrated”.

**Authors: DONE.**

38) Line 862 – Change “would be as an example” to “would be an example”.

**Authors: DONE.**

39) Line 883 – Change “expanding the knowledge of intraspecific variation” to “the expanding knowledge on intraspecific variation”.

**Authors: DONE.**

40) Line 907 – Change “increasingly” to “increasing”.

**Authors: DONE.**

41) Line 907 – Change “which we have sometimes achieved” to “which is sometimes achieved”.

**Authors: DONE.**

42) Line 926 – Change “mountain top” to “mountaintop”.

**Authors: DONE.**

43) Line 932 – Change “vegetative front” to “arboreal stratum”.

**Authors: DONE.**

44) Line 964 – Change “*Brachycephalus*’s geographic range” to “the geographic range of species in the genus *Brachycephalus*”.

**Authors: DONE.**

45) Line 970 – Change “propose” to “hypothesize”.

**Authors: DONE.**

46) Line 971 – Change “upslope” to “upward”.

**Authors: DONE.**

47) Line 976 – Change “forest” to “vegetation”.

**Authors: In this case, we are referring to the name of a forest type, based on the literature (highland dense ombrophylous forest = Floresta Ombrófila Densa Altomontana), so replacing “forest” with “vegetation” is not appropriate.**

48) Line 983 – Add “to” after “according”.

**Authors: DONE.**

49) Line 986 – Change “to Santa Catarina” to “to the state of Santa Catarina”.

**Authors: DONE.**

50) Line 999 – Change “possibly” to “possible”.

**Authors: DONE.**

51) Line 1000 – Change “conservation target species and key areas” to “target species and key areas for conservation”

**Authors: DONE.**

52) Line 1034 – Change “and, mainly, take” to “and, especially, change the status of”.

**Authors: DONE.**

53) Line 1035 – Delete “the”.

**Authors: DONE.**

54) Line 1036 – Add the word “anthropic” before “impacts”.

**Authors: DONE.**

55) Line 1043 -Change “and confirm” to “to confirm”.

**Authors: DONE.**

56) Line 1069 – Add “may have” before “occurred”.

**Authors: DONE.**

57) Line 1079 – “the lacks of” to “the apparent lack of”.

**Authors: DONE.**

58) Line 1092 - Change “improve” to “improved”.

**Authors: DONE.**

59) Line 1383 – Put the name “*Pleroma*” in italics.

**Authors: DONE.**
